# Auger electrons for cancer therapy – a review

**DOI:** 10.1186/s41181-019-0075-2

**Published:** 2019-10-11

**Authors:** Anthony Ku, Valerie J. Facca, Zhongli Cai, Raymond M. Reilly

**Affiliations:** 1 Department of Pharmaceutical Sciences, University of Toronto, Toronto, ON Canada; 2 Department of Medical Imaging, University of Toronto, Toronto, ON Canada; 3 Joint Department of Medical Imaging and Toronto General Research Institute, University Health Network, Toronto, ON Canada; 4 Leslie Dan Faculty of Pharmacy, University of Toronto, 144 College St., Toronto, ON M5S 3M2 Canada

**Keywords:** Auger electrons, ^111^In, Monoclonal antibodies, Nanoparticles, Peptides, Dosimetry, Radiolabelling, Cancer treatment, Preclinical studies, Clinical studies

## Abstract

**Background:**

Auger electrons (AEs) are very low energy electrons that are emitted by radionuclides that decay by electron capture (e.g. ^111^In, ^67^Ga, ^99m^Tc, ^195m^Pt, ^125^I and ^123^I). This energy is deposited over nanometre-micrometre distances, resulting in high linear energy transfer (LET) that is potent for causing lethal damage in cancer cells. Thus, AE-emitting radiotherapeutic agents have great potential for treatment of cancer. In this review, we describe the radiobiological properties of AEs, their radiation dosimetry, radiolabelling methods, and preclinical and clinical studies that have been performed to investigate AEs for cancer treatment.

**Results:**

AEs are most lethal to cancer cells when emitted near the cell nucleus and especially when incorporated into DNA (e.g. ^125^I-IUdR). AEs cause DNA damage both directly and indirectly via water radiolysis. AEs can also kill targeted cancer cells by damaging the cell membrane, and kill non-targeted cells through a cross-dose or bystander effect. The radiation dosimetry of AEs considers both organ doses and cellular doses. The Medical Internal Radiation Dose (MIRD) schema may be applied. Radiolabelling methods for complexing AE-emitters to biomolecules (antibodies and peptides) and nanoparticles include radioiodination (^125^I and ^123^I) or radiometal chelation (^111^In, ^67^Ga, ^99m^Tc). Cancer cells exposed *in vitro* to AE-emitting radiotherapeutic agents exhibit decreased clonogenic survival correlated at least in part with unrepaired DNA double-strand breaks (DSBs) detected by immunofluorescence for γH2AX, and chromosomal aberrations. Preclinical studies of AE-emitting radiotherapeutic agents have shown strong tumour growth inhibition *in vivo* in tumour xenograft mouse models. Minimal normal tissue toxicity was found due to the restricted toxicity of AEs mostly on tumour cells targeted by the radiotherapeutic agents. Clinical studies of AEs for cancer treatment have been limited but some encouraging results were obtained in early studies using ^111^In-DTPA-octreotide and ^125^I-IUdR, in which tumour remissions were achieved in several patients at administered amounts that caused low normal tissue toxicity, as well as promising improvements in the survival of glioblastoma patients with ^125^I-mAb 425, with minimal normal tissue toxicity.

**Conclusions:**

Proof-of-principle for AE radiotherapy of cancer has been shown preclinically, and clinically in a limited number of studies. The recent introduction of many biologically-targeted therapies for cancer creates new opportunities to design novel AE-emitting agents for cancer treatment. Pierre Auger did not conceive of the application of AEs for targeted cancer treatment, but this is a tremendously exciting future that we and many other scientists in this field envision.

## Introduction

**Fig. 1:**
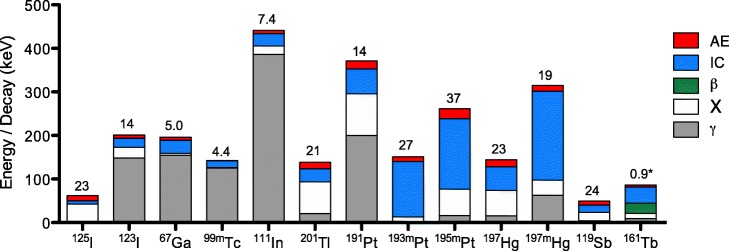
Energy contribution of γ-photons, X-rays, β-particles, internal conversion (IC) electrons, and Auger electrons (AEs) per decay event for several radionuclides. Energy estimates are based on MIRD Radionuclide Data and Decay Schemes (Eckerman and Endo 2008), and the National Nuclear Data Center for ^161^Tb (65-Terbium-161 2011). ^*^ Number of K- and L- shell Auger electrons only

Many radionuclides commonly used for imaging in nuclear medicine (e.g. ^99m^Tc, ^123^I, ^111^In, ^67^Ga; Table 1; Fig. 1) decay by electron capture (EC) and/or internal conversion (IC). As a result of these decay processes, these high atomic number elements eject a series of low energy electrons in what is referred to as the Auger effect. Although Auger electrons originating from K-shell transitions can have energy higher than 25 keV, and up to 80 keV, their yield per decay is lower than 0.1. The majority of Auger electrons (AEs) have low energy (< 25 keV), which is deposited over short nanometre-micrometre distances in tissues. This extremely short range yields high linear energy transfer (LET), which makes AEs attractive for radiation treatment of cancer, especially if they are emitted in close proximity to cell sensitive targets such as DNA and the cell membrane. These high LET electrons were first described in independent work by Lise Meitner in 1922 (Meitner 1922) and Pierre Auger in 1923 (Auger 1923). Pierre Auger later published experiments to detect electron cascades from excited atoms in his published thesis (Auger 1926). Ionisation events were visualised in these early experiments by the use of cloud chambers, where ions produced in a dense water vapour were evidenced by the condensation of a water droplet upon liberation of an electron (Wilson 1923). Pierre Auger induced what is now known as the Auger effect in noble gases excited by incident X-rays, which resulted in a primary electron ejection event and multiple electron tracks (Auger 1975).

**Table 1:** Properties of Auger electron-emitting radionuclides ^a^

Auger electrons (AEs)					Internal conversion (IC) electrons		
Radionuclide	Half-life	AEs/decay	Average AE energy per decay (keV)	Average energy per AE (keV)	IC electrons/decay	Average IC electron energy released per decay (keV)	Average energy per IC electron (keV)
^125^I	57 d	23.0	12.0	0.5	0.9	7.3	7.7
^123^I	13 h	13.7	7.2	0.5	0.2	21.0	222.6
^67^Ga	78 h	5.0	6.6	1.3	0.3	29.7	14.1
^99m^Tc	6 h	4.4	0.9	0.2	1.1	15.2	13.8
^111^In	67 h	7.4	6.9	0.9	0.2	27.9	176.1
^201^Tl	73 h	20.9	14.8	0.7	0.9	29.9	32.9
^191^Pt	2.8 d	14	17.8	1.3	304	57.1	0.2
^193m^Pt	4.3 d	27.4	10.9	0.4	3.0	126.8	42.4
^195m^Pt	4.0 d	36.6	23.1	0.6	2.8	161.4	58.1
^197^Hg	64.1 h	23.2	16.1	0.7	0.8	54.1	67.0
^197m^Hg	23.8 h	19.4	13.5	0.7	1.6	203.5	127.0
^119^Sb	38.2 h	23.7	8.9	0.4	0.8	17.0	20.2
^161^Tb^b^	6.9 d	0.9^c^	5.1^c^	5.7	1.4	36.7	26.2

^a^ The number of AEs and IC electrons were obtained from MIRD Radionuclide and Decay Schemes (Eckerman and Endo 2008)

^b^ The number of AEs and IC electrons were obtained from the National Nuclear Data Center for ^161^Tb (65-Terbium-161 2011)

^c^ Calculation based on K and L shell Auger electrons only

**Fig. 2:**
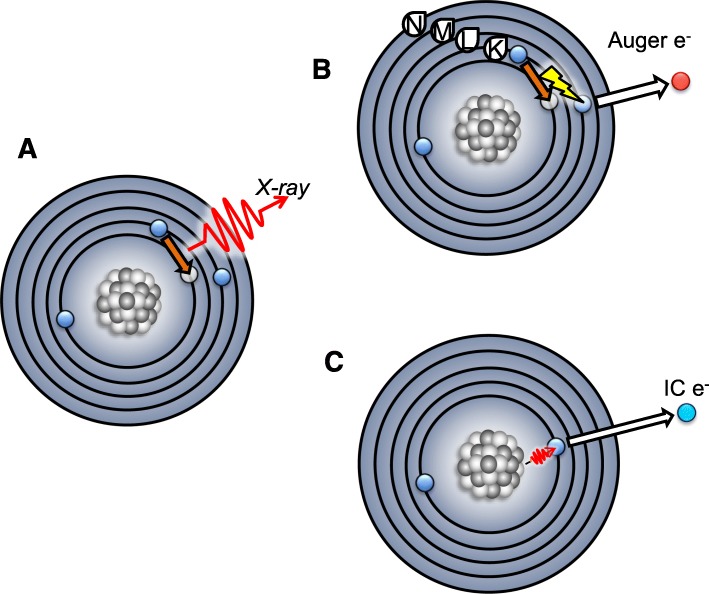
Auger electron emission can be initiated by electron capture (EC) or internal conversion (IC). In EC, protons capture an inner (K) orbital electron resulting in a primary electron vacancy. This vacancy Is filled by decay of a lower energy electron of a higher orbital (i.e. L-shell). The difference in the electronic binding energy of the two orbitals can either (**a**) result in the emission of a characteristic X-ray of energy equal to the electronic transition energy (E_L_-E_K_) or (**b**) be transferred to an electron of lower binding energy (E_b_), imparting it with kinetic energy upon ejection from the atom as an Auger electron. Progressive higher shell vacancies occur in the electron shells due to these electron transitions (open circles). **c** IC occurs in the de-excitation of unstable nuclei that impart sufficient energy to an electron to result in its ejection as an IC electron with high energy, also resulting in an inner orbital vacancy

The Auger effect describes the process in which a vacancy in an inner electron orbital (i.e. K-shell) is filled by the decay of an electron from a higher shell (i.e. L-shell) with lower binding energy. The energy difference of this transition is emitted as a characteristic X-ray (Fig. 2a), or transferred to another electron which is subsequently ejected (Fig. 2b). When the ejected electron is from the same principle energy level (i.e. L-shell), it is referred to as an Auger electron, this results in two electron vacancies within the shell, for example resulting in 2s and 2p holes. When the ejected electron is from a higher shell (i.e. M-shell), resulting in a vacancy in two different principle energy levels (i.e. holes in 2s and 3s) it is referred to as a Coster-Kronig electron, and if the resulting outer shell electron hole (i.e. 3s) is filled by an electron of the same primary shell and also ejects an electron from that same shell (i.e. holes in 3p and 3d), this is referred to as a Super Coster-Kronig electron (Howell 1992; McGuire 1975). Auger, Coster-Kronig and Super Coster-Kronig electrons are collectively referred to as Auger electrons and result from the propagation of the Auger process as a cascade of electron vacancies and ejections. The primary vacancy can be induced by an incident X-ray (as shown by Auger), or occur during the decay of unstable nuclei (radionuclides) through electron capture (EC) or internal conversion (IC) processes. EC is the process in which an inner, K-shell electron is absorbed by a proton-rich nucleus, converting the proton and absorbed electron to a neutron, maintaining the atomic mass but resulting in an excited state and a primary orbital vacancy (Intemann and Pollock 1967). IC occurs when a nucleus in an unstable excited state releases excess energy that is transferred to an inner orbital electron, overcoming its binding energy and ejecting the electron (Fig. 2c) with high kinetic energy (Choppin et al. 2002).

In this review, we describe the radiobiological properties of AEs that make them attractive for cancer therapy, discuss their radiation dosimetry and describe methods for labelling biomolecules with AE-emitting radionuclides for targeted radiotherapy of cancer. We further review the preclinical studies of Auger electrons for cancer treatment and the limited but promising clinical studies that have been performed.

## Radiobiological properties

### Particles employed in systemic radiation therapy

Key considerations in the selection of a radionuclide for systemic radiation therapy, i.e. radiation therapy using targeted biomolecules, are the energy of the emitted particles (e.g. α-particles, β-particles or AE or IC electrons), and the range of these particles in tissues. AEs have a very short range of energy deposition, offering the most precise irradiation for cancer treatment. These low energy electrons may travel up to several micrometres from the site of decay, but the majority of the electrons have sufficient energy to travel only nanometre distances (Reilly and Kassis 2010). AEs emitted from the lower electron shells, particularly propagating from K-shells, have the largest energies and travel the greatest distances, but these are produced in lower abundance than electrons ejected after transitions from the outer electron shells (M- through O-shell) which are ejected with reduced energies and travel only a few nanometres (Howell 1992). Due to the short range of AEs emitted in cascade, most result in a high LET between 1 and 23 keV/μm, which is potentially very potent for producing clustered damage in macromolecular targets of a cancer cell, particularly DNA and the cell membrane. This is different from much more energetic (MeV) β^−^ particles, which deposit most of their energy along a longer (millimetre) track length, but at the track end, some of these particles yield high LET within a nanometre range causing repairable individual DNA lesions. Additionally, most AE-emitting radionuclides emit a small number of IC electrons, with higher energies and ranges up to several millimetres, providing a longer-range effect (Eckerman and Endo 2008; Howell 1992). In comparison, α-particles deposit extremely high energy (several MeV) over 5–10 cell diameters (50–100 μm), resulting in very high LET of 50–230 keV/μm. Historically, β-particles have attracted interest for treatment of cancer due to their average long range of energy deposition of 2–10 mm in tissue, which depends on the β-particle energy. This long range causes a cross-fire effect, which irradiates not only the targeted cells but additional neighbouring cells that are within the range of the β-particles. This aids in homogenisation of the radiation dose across millimetre sized solid tumours, but can also lead to greater haematological toxicity (Carr 2004; Salem et al. 2005; Stabin et al. 2001). For example, the cross-fire effect of the β-particles emitted by ^177^Lu or ^90^Y (maximum range of 1.7 or 11 mm in tissue, respectively) (Lai et al. 2017), radionuclides that have been complexed to monoclonal antibodies for radioimmunotherapy (RIT) of cancer, contributes to dose-limiting non-targeted hematopoietic toxicity (Vallabhajosula et al. 2005) through irradiation of bone marrow cells adjacent to skeletal regions of nonspecific uptake, or skeletal metastases, as well as circulating radioactivity perfusing the bone marrow.

### Targets for cytotoxicity from AEs

**Fig. 3:**
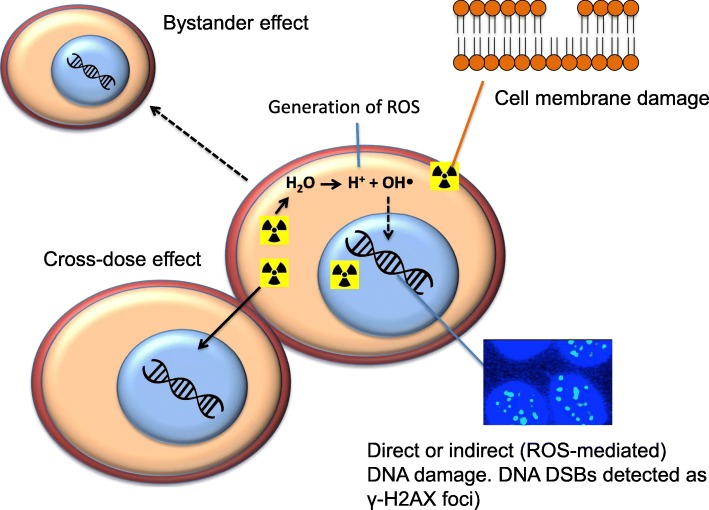
Modes of cell death caused by Auger electron (AE) emission. AEs may cause DNA double-strand breaks (DSBs) by a direct effect or through an indirect effect mediated by hydroxyl free radicals (ROS) due to interaction with water molecules. AEs may also cause cell membrane damage leading to cell death. There is a localised short-range “cross-dose” effect of AEs on cancer cells which are directly adjacent to targeted cells, and a longer range “bystander” effect on more distant cells

Radiation induced cell death may proceed via several different mechanisms (Fig. 3). Direct DNA damage may be caused by traversal of the DNA duplex by high LET particles such as AEs or α-particles. Alternatively, indirect damage to DNA may be inflicted by reactive hydroxyl radicals (ROS) that are generated by interaction of AE, α-particles, β-particles, γ-photons or X-rays with water molecules and consequent radiolysis. The cascade of electrons and resultant local generation of free radicals may result in the formation of a concentrated region of macromolecular damage, which in the case of a DNA target can result in multiple and complex DNA breaks. Unrepaired damage can then result in cell death by several pathways, e.g. apoptosis or mitotic catastrophe (Haefliger et al. 2005; Kriehuber et al. 2004a; Urashima et al. 2006). DNA is considered the main target for causing radiation-induced cell death and indeed, the greater the unrepaired DNA damage the higher the incidence of lethality (Chan et al. 1976; Schneider and Whitmore 1963). Radiation-induced DNA double strand breaks (DSBs) are recognised by the protein kinase ataxia telangiectasia mutated (ATM), and through ATM/p53 pathways result in cell cycle arrest or apoptosis (Ismail et al. 2005). However, tumour cells of epithelial origin commonly have deficient pro-apoptotic pathways and are mainly killed through mitotic catastrophe as a consequence of attempting to enter mitosis with damaged DNA (Eriksson and Stigbrand 2010). Phenotypic features of cells entering pre-mature mitosis are aberrant chromosomes, multiple nuclei and micronuclei (Bhattathiri et al. 1998).

Since DNA has historically been considered the principal target for radiation treatment of cancer (Burdak-Rothkamm and Prise 2009), the emission of AEs in or near the cell nucleus, and especially in close proximity to the DNA greatly amplifies the lethality of these electrons, particularly taking into account the subcellular range of these electrons (Faraggi et al. 1994; Fasih et al. 2012; Hoang et al. 2012). Indeed when decaying in close proximity to DNA, AEs emitted by ^125^I exhibited superior cell killing properties than the longer-range β-particles emitted by ^131^I (Chan et al. 1976). Kassis and Adelstein showed that the AEs emitted by ^125^I were most lethal to Chinese hamster V79 lung fibroblasts when emitted by ^125^I-iododeoxyuridine (^125^I-IUdR), a nucleoside analogue that is incorporated into DNA, than with ^125^I-iododihydrorhodamine which is localised in the cytoplasm (Kassis et al. 1987). Relative biological effectiveness (RBE) describes the ratio of the effectiveness of one form of radiation compared to another, with X-rays or γ-radiation often used for comparison. An RBE = 1 indicates that the radiation has equivalent biological effects (e.g. cytotoxicity) as X-rays or γ-radiation. For deterministic effects, AE have exhibited RBE in the range of 2–5 (Freudenberg et al. 2014; Kriehuber et al. 2004b; Regulla et al. 2002; Yasui et al. 2001). The high lethality of AEs emitted near the nucleus is apparent in a comparison of RBE considering whole cell or nuclear targeting. Cellular dose calculations for the AE-radionuclides ^99m^Tc and ^123^I in rat thyroid PC Cl3 cells, assuming a whole cell or nuclear target for dose calculations, resulted in an increased RBE from 0.75 to 2.18, for ^99m^Tc and from 1.87 to 3.43 for ^123^I (Freudenberg et al. 2014). The enhanced efficacy of AE in proximity to DNA is illustrated in one report that showed that the RBE for ^125^I incorporated into DNA was almost 8, and that these low energy AEs were equivalently cytotoxic as the 5.3 MeV α-particles emitted by ^210^Po (Rao et al. 1989). However, if AEs were emitted in the cytoplasm, the RBE was ~ 1, and similar to low LET radiation such as X-rays (Rao et al. 1990). A modelling study of dose deposition in the DNA duplex revealed higher energy deposition by the AEs emitted by ^125^I than 5 MeV α-particles, and that even at a distance in DNA as short as 5 base pairs from the AE emission, the energy deposition was reduced by a factor of 10 (Charlton 1986). Some insightful studies employed triplex-forming oligonucleotides or minor groove-binding ligands that hybridised to DNA labelled with the AE-emitters ^125^I or ^111^In, to illustrate the distant-dependent effects of AEs on causing DNA double-strand breaks (Karamychev et al. 2000; Lobachevsky et al. 2008; Panyutin and Neumann 1997). Panyutin and Neumann (1997) demonstrated using ^125^I-labelled DNA triplex forming oligonucleotides that the probability of DNA strand breaks was strongly correlated with distance from the position of radionuclide decay, with 90% of DNA breaks found within 10 base pairs (Panyutin and Neumann 1997). Free radical scavenging studies that employed dimethylsulfoxide (DMSO) to eliminate indirect DNA damage, illustrated that beyond a critical distance, the DNA DSBs induced by AEs were mediated mainly by indirect effects caused by oxygen free radicals (Balagurumoorthy et al. 2012; Lobachevsky et al. 2008; Panyutin and Neumann 1997).

### Detection of DNA damage

Interaction of AEs or their induced reactive oxygen species (ROS) with DNA can cause complex and multiple DSB (Fig. 3). The accumulation of unrepaired DNA DSB is negatively correlated with cell survival (Cai et al. 2008) and the misrepair of these breaks from damaging AE can result in chromosomal aberrations, similarly associated with reduced cell survival (Beckmann et al. 1993; Woo et al. 1989). Assays for DSBs induced in cancer cells by AEs are thus useful for studying their cytotoxicity. Common assays used for radiation-induced DNA DSBs include γH2AX, comet assay, and pulsed-field gel electrophoresis (PFGE). In response to DSBs caused by ionizing radiation, the histone H2AX is rapidly phosphorylated (γH2AX) and accumulates at these sites of DNA damage (Mah et al. 2011). The phosphorylation of H2AX can be detected within one minute after exposure to ionizing radiation and reaches maximal levels within 10–30 min (Rogakou et al. 1998). γH2AX can be imaged by confocal immunofluorescence microscopy of the nucleus of cells and appears as discrete foci, which are a measure of radiation induced DNA DSB (Cai et al. 2009). Immunofluorescent imaging of γH2AX foci has proven useful in the study of AE-mediated DNA damage in cancer cells (Cai et al. 2008; Mah et al. 2011; Piron et al. 2014; Sedelnikova et al. 2002; Yasui et al. 2007). A direct correlation was found between the number of radionuclide decays and the number of foci observed for human glioblastoma SF-268 and fibrosarcoma HT-1080 cells treated with ^125^I-IUdR, demonstrating a similar response in two different cancerous cell lines (Sedelnikova et al. 2002).

The single cell gel electrophoresis assay (SCGE) or comet assay, allows assessment of DNA damage at the single cell level. Cells are embedded in agarose and lysed with detergents at high concentrations of salt. Application of an electric field resolves fragmented DNA as a “tail” beyond the large, supercoiled, undamaged DNA “head” (Olive and Banath 1993). The comet assay has been useful to assess DNA damage caused by AEs (Haines et al. 2001; Hoyes et al. 2001; Olive and Banath 1993; Pedraza-López et al. 2000; Piron et al. 2014; Reske et al. 2007). For example, the comet assay was used to show increased DNA damage caused by ^99m^Tc-hexamethylpropylenamineoxime (HMPAO) over ^99m^Tc gentisic acid when internalised into the cytoplasm or remaining bound to the cell membrane of lymphocytes, respectively (Pedraza-López et al. 2000). It was also used to demonstrate extensive DNA damage caused by ^125^I-IUdR or ^123^I-4′-thio-2′-deoxyuridine (^123^I-ItdU) incorporated into DNA (Olive and Banath 1993; Reske et al. 2007).

Pulsed field gel electrophoresis (PFGE) allows for improved separation of large DNA fragments beyond the capabilities of traditional gel electrophoresis by employing an alternating electric field. PFGE is useful for analysis of larger samples of DNA from a high number of cells, contrasting it from single cell gel electrophoresis. PFGE has been used to examine DSB caused by AEs from ^125^I-IUdR in hamster V79-379A cells (Elmroth and Stenerlöw 2005) and Chinese Hamster Ovary (CHO) cells (Iliakis et al. 1991).

### Cross-dose, bystander effects and cell membrane damage

The cross-fire effect, most commonly attributed to β-particles, describes the irradiation of cells distant from those harbouring the radionuclide due to the long (several millimetres) range of these particles. Despite the short nanometre-micrometre range of AEs, there is a local cross-dose effect of AE-emitting radionuclides that deposits dose in tumour cells that are immediately adjacent to cells in which the radionuclide decays (Cai et al. 2010) (Fig. 3). This is mediated by the several micrometre range of some higher energy AEs and IC electrons. In addition, tumour cells with lethal DNA damage caused by radiation may release mediators that cause the death of distant non-irradiated cells through the bystander effect (Mothersill et al. 2018) (Fig. 3). The bystander effect has been observed for AEs *in vitro* in media transfer experiments in which growth medium from donor cells exposed to ^123^I-metaiodobenzylguanidine (^123^I-MIBG) was transferred to non-irradiated recipient cells causing decreased clonogenic survival of these cells (Boyd et al. 2006; Paillas et al. 2016). Diminished clonogenic survival and increased numbers of γH2AX foci in HCT116 colon cancer cells were observed by media transfer experiments following exposure of donor cells to ^125^I-labelled anti-epidermal growth factor receptor (EGFR) monoclonal antibodies (mAb) (Paillas et al. 2016). Other studies have shown greater inhibition of tumour growth in mice inoculated with a mixture of non-irradiated cells and pre-irradiated cells compared to non-irradiated cells alone, demonstrating an AE-mediated bystander effect *in vivo* (Xue et al. 2002).

Due to the short range of most AEs, considerable attention has been focused on delivery of AE-emitting radionuclides to the nucleus or DNA (historically considered the primary cellular target of radiation damage) of tumour cells to maximise their cytotoxic effects. However, it has been shown that internalisation into cancer cells and delivery to the cell nucleus is not obligatory for cell killing, and that the lethal effects of AEs may be induced indirectly by free radical-mediated pathways (Goddu et al. 1996; Narra et al. 1995). Targeting the cell membrane has been proven to be an effective strategy for killing cancer cells with AEs (Paillas et al. 2016; Pouget et al. 2008; Santoro et al. 2009) (Fig. 3). In *in vitro* experiments, non-internalising ^125^I-anti-carcinoembryonic (CEA) mAbs bound to the surface of HCT116 colon cancer cells generated ROS that caused re-organisation of lipid rafts and activated receptor-mediated cell signalling pathways (ERK1/2, AKT, p38/JNK) and several phosphorylated protein mediators of Ca^2+^ levels (phospholipase C-γ and proline-rich tyrosine kinase 2 and paxillin) (Paillas et al. 2016). Cell membrane damage further induced γH2AX foci in the nucleus of donor cells exposed to ^125^I-anti-CEA mAbs and in recipient, non-exposed cells through a bystander effect. This study further revealed that DNA damage was quite homogeneous in CEA-positive A431 tumours in mice administered ^125^I-anti-CEA mAbs, despite radioactivity being localised mainly at the periphery of the tumour, suggesting a local bystander effect on non-targeted cells that could be mediated by damage to the cell membrane of targeted tumour cells (Paillas et al. 2016). ^125^I-labelled anti-CEA 35A7 was also found to be effective *in vivo* for treatment of small peritoneal tumours in mice, illustrating that internalisation and nuclear importation are not always required for the use of AEs for cancer therapy (Santoro et al. 2009). These findings are promising since they extend the targets for AE radiotherapy of cancer to non-internalising cell surface antigens overexpressed on tumour cells that are recognised by mAbs or other ligands.

## Dosimetric properties

### Organ and cellular dosimetry of AEs

Radionuclides that emit AEs also release γ-rays and X-rays and IC electrons. AEs have energies from a few eV to tens of keV, and ranges in soft tissue from a few nm up to 100 μm, while IC electrons have higher energies (tens to hundreds keV) and ranges (tens of μm to several mm). In contrast, γ-rays and X-rays are penetrating and travel much farther, mostly much longer than cm distances, except that for ultra-soft X-rays, the range can be as low as a few μm (Eckerman and Endo 2008; Berger et al. 2005; Hubbell and Seltzer 2004). Due to the diverse radiations and energy deposition distances and the dimension of critical targets, which range from single cells and subcellular compartments, to tumour masses and normal organs, in order to understand the whole picture of dosimetry for AE emitting radionuclides, both organ and cell doses should be considered (Bolch et al. 2009; Goddu et al. 1997; Loevinger et al. 1988; Roeske et al. 2008; Stabin 2006; Thierens et al. 2001; Vaziri et al. 2014). Organ dosimetry estimates the absorbed dose at the whole organ level, to which photons are the main contributor when the target organ is different than the source organ. Electrons play the prominent role when the source and target organs are the same, i.e. radioactivity localised in a source organ deposits energy in that same organ. However, when we zoom in one million times to examine the absorbed dose at the subcellular level, we need cellular dosimetry. This is especially the case for short range radiation such as AEs. Cellular dosimetry is the study of energy deposition at the cellular level, which considers both the cellular targets and sources (e.g. cell, cell membrane, cytoplasm and nucleus) as a quantitative means of understanding the biophysical interactions of radiation with matter (Goddu et al. 1997; Roeske et al. 2008; Vaziri et al. 2014).

The Medical Internal Radiation Dose (MIRD) schema is the gold standard to estimate the internal absorbed dose from radiopharmaceuticals. It can be described by eq. 1:

1 $$ \mathrm{D}\left({\mathrm{r}}_{\mathrm{T}},{\mathrm{T}}_{\mathrm{D}}\right)=\sum \limits_{r_S}{\widetilde{\mathrm{A}}}_{\left({\mathrm{r}}_{\mathrm{s}},{\mathrm{T}}_{\mathrm{D}}\right)}{\mathrm{S}}_{\left({\mathrm{r}}_{\mathrm{T}}\leftarrow {\mathrm{r}}_{\mathrm{S}}\right)} $$

where D (r_T_,T_D_) is the mean absorbed dose to the target region (r_T_) over a dose-integration period (T_D_), from a radionuclide distributed uniformly within a source region (r_S_). Ã (r_S_, T_D_) is the time-integrated radioactivity in r_S_ over T_D_, while S (r_T_ ← r_S_) is the absorbed dose in r_T_ per radioactive decay in r_S_. The MIRD schema is applicable to both organ and cellular dosimetry (Dewaraja et al. 2012; Goddu et al. 1997; Siegel et al. 1999; Vaziri et al. 2014).

To calculate Ã (r_S_, T_D_), it is necessary to measure the source radioactivity at different time points to produce the time-radioactivity curve, followed by integration of this curve over T_D_. For clinical studies using AE emitting radionuclides such as ^111^In (Fisher et al. 2009; Vallis et al. 2014), ^99m^Tc (Ocampo-Garcia et al. 2017) and ^123^I (Chin et al. 2014), planar imaging, SPECT/CT, non-imaging whole body radioactivity monitoring, or tissue sampling (e.g. blood and urine) may be used to assay the source radioactivity (Siegel et al. 1999). For animal studies, besides these imaging approaches used in the clinic, the source radioactivity is most commonly quantified by harvesting samples of source organs after euthanising animals at selected time points, then measuring the radioactivity in these tissue samples and the weight of the tissue sample to calculate the radioactivity per gram of tissue (Cai et al. 2016; Razumienko et al. 2016). Since organ dosimetry is based on Ã (r_S_, T_D_) in the entire organ, these tissue concentrations are multiplied by the organ weights to obtain the radioactivity per organ. For cellular dosimetry, cells are incubated with the radiopharmaceutical for different times, then cell fractionation is performed to separate the radioactivity bound to the cell membrane, internalised into the cytoplasm or imported into the nucleus, followed by measuring the radioactivity in each of these fractions by γ-counting (Ngo Ndjock Mbong et al. 2015). The selection of time points depends on both the biological (T_b_) and physical (T_p_) half-lives of the radiopharmaceuticals, which can be summarised by the effective half-life (T_e_):

$$ {\mathrm{T}}_{\mathrm{e}}={\mathrm{T}}_{\mathrm{b}}\ {\mathrm{T}}_{\mathrm{p}}/\left({\mathrm{T}}_{\mathrm{b}}+{\mathrm{T}}_{\mathrm{p}}\right) $$

**Fig. 4:**
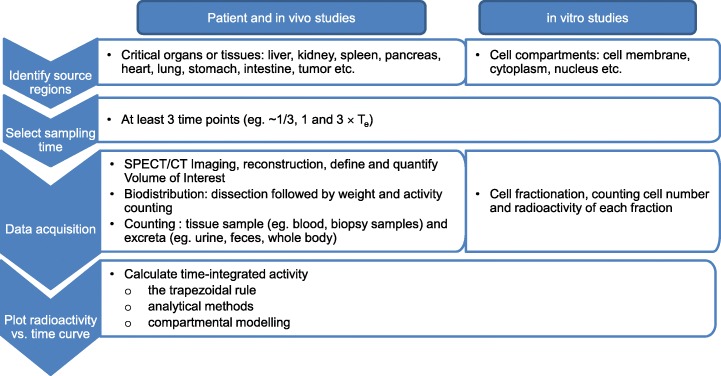
Outline of the steps to acquire time-integrated radioactivity (Ã) *in vivo* in tumours and normal organs, and *in vitro* in cancer cells for macro- or micro-dosimetry estimates, respectively

At least 3 time points (e.g. ~ 1/3, 1 and 3 × T_e_) should be selected for each exponential term of the curve (Siegel et al. 1999). For example, 5 time points at 0, 4–6 h; 1, 3, 6 days post-injection (p.i.) of ^111^In-ibritumomab tiuxetan (Zevalin) mAbs were selected for quantitative SPECT to estimate the organ doses in a study of 10 lymphoma patients (Fisher et al. 2009). A shorter time period was used for estimating the doses from ^111^In-DTPA-human epidermal growth factor (^111^In-DTPA-hEGF) in 15 patients with EGFR positive breast cancer using whole body planar images acquired at 1, 4–6, 24 and 72 h p.i., since radiopeptides are eliminated more rapidly than mAbs (Vallis et al. 2014). Whole-body images were obtained at 20 min, 2, 6, 24 h post injection of ^99m^Tc-EDDA/HYNIC-Tyr (3)-octreotide to estimate the radiation doses in 4 healthy individuals (Ocampo-Garcia et al. 2017). The choice of collimators and scan time, as well as attenuation and scatter correction used in image reconstruction all have effects on the accuracy of image quantification (Dewaraja et al. 2012; Siegel et al. 1999). Sub-organ regional dosimetry for AE-emitting radionuclides is desirable for critical organs such as the kidneys and liver, as well as the tumour due to the spatial non-uniformities of radioactivity distribution (Dewaraja et al. 2012). This is particularly true for the kidneys, since it has been found that the sub-organ distribution greatly influences the nephrotoxicity of radiopeptides, due to differences in the range of the β-particles emitted by ^90^Y or ^177^Lu and the AEs emitted by ^111^In and the distance between radioactivity localised in the renal tubules and the more radiosensitive glomeruli (Konijnenberg et al. 2004). For patients, it might be challenging to acquire SPECT/CT with sufficient resolution to accurately quantify radioactivity at different regions in critical normal organs or a tumour. The spatial resolution of clinical SPECT is 5–10 mm (Dewaraja et al. 2012). Recently resolutions of 2.5 and 1.6 mm were reported in images of a phantom using a multi-pinhole clinical (Chen et al. 2018) or preclinical (Massari et al. 2019) SPECT system, respectively. However, for animal studies, it is possible to perform autoradiography in combination with immunohistochemical staining of excised tissues *ex vivo* to understand the heterogeneous radioactivity distribution in different regions of tumours and normal organs (Lee et al. 2010). In one study, to understand the effect of heterogeneous radioactivity distribution in the tumour on the treatment response, tumour cell spheroids were cultured and incubated with ^111^In-DTPA-hEGF or ^111^In-DTPA-trastuzumab, then fixed, frozen, and cryo-sectioned (Falzone et al. 2018, 2019). One section was used for micro-autoradiography to visualise the radioactivity distribution and the adjacent section was stained for γH2AX to assess DNA damage. Ã (r_S_, T_D_) can be calculated from the area under the curve (AUC) via numerical methods such as the trapezoidal rule, or analytical methods by fitting the time-radioactivity curve to a sum of exponentials, followed by integration from time 0 to T_D_. If the radioactivity of r_S_ cannot be directly measured, its time-radioactivity curve may be derived via compartmental modelling of the measured data of other regions with which r_S_ is physiologically interacted (Siegel et al. 1999). Recently a robust biodistribution reporting and publication standard were proposed (Kesner and Bodei 2018) and an International Atomic Energy Agency (IAEA) Radiotracer Biodistribution Template (IAEA RaBiT) allowing detailed and standardised input of biodistribution data (Kesner et al. 2017) for dosimetry estimates is free to download (IAEA Radiotracer Biodistribution Template (RaBiT) n.d.). Figure 4 outlines the steps of acquiring Ã *in vivo* and *in vitro* for dosimetry estimates.

**Fig. 5:**
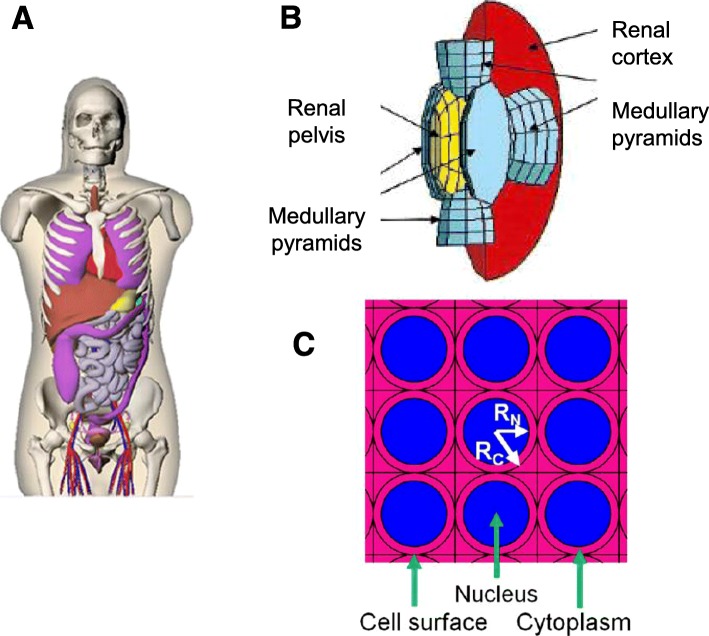
Representative phantoms used in Monte Carlo Simulation for S-values of human organs, important kidney regions and subcellular compartments in a hexahedrally closely packed monolayer of cells: (**a**) a voxel-based realistic female phantom applied by OLINDA/EXM version 2.0 software (Stabin and Siegel 2018). **b** Kidney created using geometric shapes to represent renal pelvis, renal cortex and medullary pyramids (Bouchet 2003). **c** Cellular phantom with cell and cell nucleus represented by co-centric spheres with cell and nucleus radius of R_C_ and R_N_, respectively (Cai et al. 2017)

S-values depend on the physical properties of the radionuclides, and the size and geometry of source and target regions. They can be calculated either via Monte Carlo simulation or analytical methods in the case of cellular dosimetry. Figure 5 shows representative models of reference man (A), the kidney (B), or cells (C) used in Monte Carlo Simulation of S-values (Stabin and Siegel 2018; Bouchet et al. 2003; Cai et al. 2017). Monte Carlo code used to calculate S-values includes MCNP6 (Di Maria et al. 2018), PENELOPE (Falzone et al. 2017; López-Coello et al. 2019), Geant4 (López-Coello et al. 2019) and GATE (López-Coello et al. 2019). They all allow event-by-event simulation of electron and photon transport. Though Monte Carlo code is mostly used in condensed history mode for organ dosimetry, event-by-event simulation is more accurate for cellular dosimetry and is required for electrons with energies lower than 1 keV (Tajik-Mansoury et al. 2017). Monte Carlo simulation is more accurate but also more time consuming and computationally demanding. However, S-values for organs of several human phantoms, mice and rat tissues, cells and cell compartments are available in MIRD Pamphlets (Bolch et al. 1999; Bouchet et al. 1999, 2003; Goddu et al. 1997; Vaziri et al. 2014), ICRP reports (Mattsson et al. 2015) and numerous other publications. IDAC-Dose2.1 (Abuqbeitah et al. 2018; Andersson et al. 2017), an internal dosimetry computer program for diagnostic nuclear medicine based on the ICRP adult reference voxel phantoms (both male and female), is free software (IDAC-Dose2.1 n.d.) that allows calculation of the absorbed doses based on measured Ã. When human dosimetry is extrapolated from animal experiments, the radioactivity in normal organs of a human could be estimated from that in the respective organs of animals using the %kg/g method (Kirschner et al. 1975): (%ID/organ)_human_ = [(%ID/organ)_animal_ × (animal body weight/animal organ weight) × (human organ weight/human body weight). Another free software package, MIRDcell V2.1 (MIRDcell, a Multicellular Dosimetry Tool n.d.), a Multicellular Dosimetry tool, calculates cellular doses where the source is defined as the whole cell, cell surface, cytoplasm and nucleus and the target as the whole cell, nucleus or cytoplasm with cell and nucleus radius, as well as distance between cells adjustable in 1 μm increments. The cell and nucleus shape can only be defined as concentric spheres. The multicellular geometry can be either single cell, 1-D (dimensional) cell pair, 2-D colony or a 3-D cluster with sphere, rod, cone or ellipsoid shape. Cell labelling can be selected as uniform, log-normal or normal distribution (Vaziri et al. 2014). S values calculated with MIRDcell correlated well with those by Monte Carlo simulation, however, the relative error can be as high as 68%, depending on the source and target sizes as well as particle ranges and simulation codes. Comparable scoring size and particle range leads to the biggest differences between different Monte Carlo simulations (Tajik-Mansoury et al. 2017). Olinda/EXM (Stabin et al. 2005) is more widely used than IDAC-Dose2.1, but is not free software. Olinda/EXM 1.0 includes 10 phantom models; adult male, adult female, 1, 5, 10 and 15-year old, newborn, 3-, 6-, 9-month pregnant woman as well as models of the prostate gland, peritoneal cavity, spheres (used for tumours), head and kidneys. The anthropomorphic phantoms define the body and its organs with geometrical shape. Olinda/EXM 2.0 replaces the above phantoms with realistic, non-uniform rational B-splines (NURBS) voxel-based models and uses updated decay data. It also includes phantoms for the mouse, rat and dog. IDAC-Dose2.1, MIRDcell V2.1 and Olinda/EXM all use radiation spectra obtained via the (MIRD) RADTABS program. Nuclear Decay Data in the MIRD Format (https://www.nndc.bnl.gov/mird/) also produces continuingly evaluated radiation spectra of radionuclides including Auger electron emitters. However, it should be noted that the (MIRD) RADTABS program provides detailed Auger and Coster-Kronig electron spectra while the National Nuclear Data Centre only lists average energies and yields of Auger-K and Auger-L electrons. Recently it was reported that the yield of Auger, CK electrons from (MIRD) RADTABS was consistently higher than from the newly developed stochastic atomic relaxation model BrIccEmis (Falzone et al. 2017). The difference in spectra had a significant impact on the S-values only at the nanoscale.

Due to the subcellular and cellular range of AEs, for a single cell, S_N ← N_ > > S_N ← Cy_ > S_N ← CS_. For a monolayer of cells or a cell cluster, this trend remains the same, but the difference becomes smaller due to the contribution of cross-dose (Cai et al. 2010). Although nuclear DNA is the most sensitive target for radiation, recent studies show that radiation damage to mitochondria and cell membrane leads to apoptosis or cell death (Bavelaar et al. 2018; Kirkby and Ghasroddashti 2015; Pouget et al. 2018). A Monte Carlo mitochondrial dosimetry study on the β- and γ-emitter, ^131^I showed 2 order higher S-values for the mitochondrial source to mitochondrial target compartment compared to the nucleus to nucleus (Carrillo-Cázares and Torres-García 2012). It would be interesting to calculate S-values for the cell membrane source to cell membrane target and mitochondrial source to mitochondrial target for AE-emitting radionuclides, for correlation with the cytotoxic effects.

The dosimetry of AEs is important for understanding their effects on radiation sensitive targets in cells, and for predicting their effectiveness for cancer treatment as well as any potential toxicities to critical organs (e.g. red marrow, kidneys, liver and spleen). Preclinically, cellular dosimetry may be estimated based on subcellular distribution of radioactivity measured by cell fractionation studies, and organ dosimetry may be estimated in mouse tumour xenograft models by organ biodistribution studies which can be combined with *ex vivo* cell fractionation to measure subcellular distribution (Costantini et al. 2007). Clinically, however, there are challenges in estimating subcellular doses from AEs, since information is not available on the subcellular radioactivity distribution. Nonetheless, tumour and normal organ dosimetry is feasible. It should nevertheless be realised that there are limitations to tumour dosimetry in predicting the therapeutic response from targeted radiotherapeutic agents in patients, due to heterogeneous tumour uptake, inaccuracies in dose estimates based on the imaging data, and radiobiological factors including the sensitivity of tumours to exponentially decreasing low dose rate radiation emitted by AEs and other forms of radiation used (in contrast to external radiation treatment which is much higher dose rate radiation). Organ dosimetry has nonetheless been found to be quite accurate in predicting normal tissue toxicity to the bone marrow and the kidneys in patients receiving targeted radiotherapeutic agents (Chalkia et al. 2015; Sarnelli et al. 2017; Strigari et al. 2014). Absorbed doses are only the first step to understand the dose-effect relationships. The microenvironments and radiosensitivity of the tumour and normal tissues, radiation chemistry reactions and radiobiological effects such as the bystander effect induced by physical dose absorption all contribute to the outcomes (Pouget et al. 2018; Ma et al. 2019; Dong et al. 2019).

## Radiolabelling approaches

**Fig. 6:**
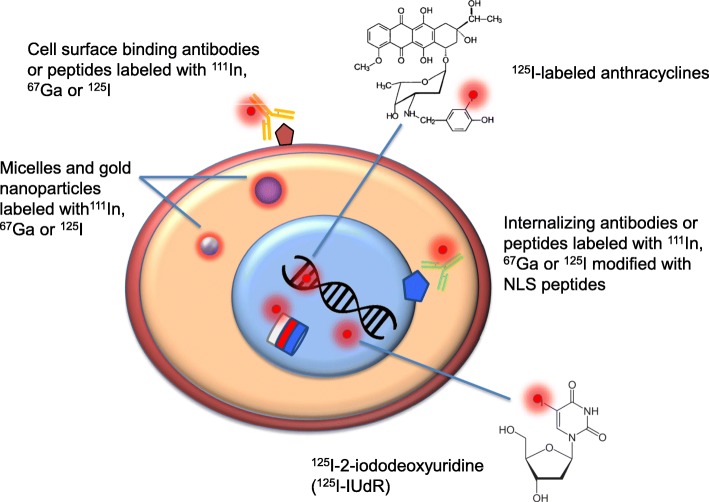
Examples of targeting vehicles for delivery of Auger electron (AE)-emitting radionuclides to cancer cells. Non-internalising monoclonal antibodies (mAbs) or peptides bind to cell-surface receptors and may kill cells through damaging the cell membrane. Internalising mAbs or peptides may be transported to the cell nucleus by appending nuclear localisation sequence (NLS) peptides or by an endogenous NLS present in some cell-surface receptors (e.g. EGFR or HER2) or combine internalising, endosomal escape and nuclear localizing sequences (modular nanotransporters). Nuclear localisation causes lethal DNA double-strand breaks (DSBs). Micelles or gold nanoparticles may be modified with mAbs or peptides to target cell surface receptors or are internalised non-specifically into cancer cells by endocytosis. ^125^I-labelled anthracyclines diffuse into cancer cells and intercalate into DNA, while ^125^I-2-iododeoxyuridine (^125^I-IUdR) is taken up by nucleoside transporters and incorporated into DNA. These agents cause lethal DNA DSBs

To achieve selective delivery of AE-emitting radionuclides to tumours for cancer treatment, these radionuclides must be attached to targeting ligands such as monoclonal antibodies (mAbs), or peptides that recognise cell surface receptors displayed on cancer cells (Fig. 6). In some cases, nanoparticles that incorporate polymers that complex AE-emitters and are modified on their surface with targeting ligands have also been used (Cai et al. 2016; Fonge et al. 2009; Hoang et al. 2012, 2013). Radiolabelling of mAbs and peptides with AE-emitters may be performed by radioiodination (^125^I or ^123^I), or by modification with bifunctional metal chelators (e.g. DTPA) that complex radiometals (^111^In, ^67^Ga, ^64^Cu; Table 1). Radioiodination requires oxidation of ^125^I or ^123^I from a valence state of − 1 as sodium iodide to + 1 for electrophilic substitution onto tyrosine amino acids in mAbs or peptides (Reilly 2007). Iodogen® (1,3,4,6-tetrachloro-3α,6α-diphenyl-glycoluril; Fraker and Speck Jr 1978) is the most commonly used oxidising reagent. However, since radioiodination efficiency is typically low (40–60%), radioiodinated mAbs and peptides must be purified post-labelling to remove free ^125^I or ^123^I. This does not allow kit formulation for preparing the radiopharmaceutical. Another disadvantage is that radioiodinated proteins are deiodinated *in vivo* by deiodinases, which are ubiquitous in the body and function to deiodinate thyroid hormones (Reilly 2007). In contrast, mAbs and peptides labelled with radiometals via chelation are relatively stable *in vivo*, and complexation of radiometals by chelators is very efficient (> 90–95%) enabling kit formulation. We previously reported the design of a kit for labelling DTPA-hEGF with ^111^In for AE radiotherapy of EGFR-positive breast cancer (Reilly et al. 2004). The most commonly used chelators for complexing AE-emitting radiometals are DTPA (diethylenetriaminepentaacetic acid), DOTA (1,4,7,10-tetraazacyclododecane-1,4,7,10-tetraacetic acid) and NOTA (1,4,7-triazacyclononane-1,4,7-triacetic acid). DOTA and NOTA are most suitable for labelling mAbs and peptides with ^67^Ga or ^64^Cu, but DOTA may also be used for ^111^In-labelling (Reilly 2007). DOTA and NOTA are macrocyclic chelators that provide a more stable radiometal complex than DTPA, but this causes slower labelling kinetics which requires mild heating of the DOTA-conjugated mAbs to 42 °C and longer incubation times (Razumienko et al. 2016). Peptides are more heat-stable than mAbs and thus DOTA-peptide conjugates may be heated up to 95 °C to achieve rapid and efficient complexation of radiometals (Brom et al. 2012). Several bifunctional chelators have been synthesised with a chemically-reactive group positioned on a side-chain for conjugation to mAbs to preserve the most stable octadentate radiometal-chelator complex. These include p-isothiocyanatobenzyl DTPA (p-SCN-Bn-DTPA), p-SCN-Bn-DOTA, and p-SCN-Bn-NOTA (Macrocyclics, Inc., Plano, TX, USA). The p-isothiocyanate group forms a thiourea linkage with ε-amino groups on lysines on the mAbs. Peptides may be assembled by solid-phase peptide synthesis which allows a chelator to be positioned at a specific location in the sequence to complex radiometals (Lever et al. 2019).

An important consideration for cancer treatment with AEs is the specific activity (SA) of the radiopharmaceuticals, since this defines the amount of radioactivity delivered to a single cancer cell per receptor-recognition event. At the SA usually achieved for labelling mAbs with ^111^In (< 1 MBq/μg), there may be as few as 1 in 50 antibody molecules that are radiolabelled, which limits their effectiveness for killing cancer cells, especially cells that have low-moderate target receptor expression (Ngo Ndjock Mbong et al. 2015). A strategy which we have explored to increase the SA of trastuzumab labelled with ^111^In is conjugation of the antibodies to G4 polyamidoamine (PAMAM) dendrimers derivatised with multiple DTPA (Chan et al. 2013). This approach increased the SA by 100-fold compared to conjugation directly with DTPA and increased the cytotoxicity *in vitro* of these radioimmunoconjugates (RICs) on HER2-positive breast cancer cells by up to 9-fold. Another approach is conjugation of the mAbs with metal-chelating polymers (MCPs) that present multiple DOTA chelators for ^111^In (Aghevlian et al. 2018; Ngo Ndjock Mbong et al. 2015). Conjugation of trastuzumab to MCPs presenting 24–29 DTPA chelators increased the SA of trastuzumab labelled with ^111^In by 90-fold compared to trastuzumab conjugated to DTPA and increased the cytotoxicity of these RICs *in vitro* on HER2-positive breast cancer (BC) cells, including cells with low-moderate HER2 expression (Ngo Ndjock Mbong et al. 2015). Panitumumab modified with an MCP that presented 13 DOTA chelators increased the SA for labelling with ^111^In to > 70 MBq/μg (Aghevlian et al. 2018).

Nanoparticles may be labelled with AE emitters by incorporation of polymers that present DTPA or DOTA for complexing ^111^In. For example, ^111^In-labelled block copolymer micelles (BCMs) modified with EGF to target EGFR-positive breast cancer cells were assembled by incorporating a DTPA-polyethylene glycol (PEG)-b-polycaprolactone (PCL) block polymer into these micelles (Fonge et al. 2009). ^111^In-labelled BCMs modified with trastuzumab to target HER2-positive breast cancer cells were similarly constructed (Hoang et al. 2012). We labelled gold nanoparticles (AuNPs) modified with trastuzumab by attaching ^111^In-DTPA-PEG polymers to the surface of the AuNPs via a gold-thiol linkage (Cai et al. 2016).

## Preclinical studies

### DNA-binding Radiotherapeutic agents

Since AEs are most damaging to DNA and lethal when emitted in close proximity to DNA, the pioneering work on AEs for cancer treatment was performed by Kassis and Adelstein using ^125^I- or ^123^I-5-iodo-2-deoxyuridine (IUdR) (Kassis et al. 1987; Makrigiorgos et al. 1989). ^125^I/^1^^23^I-IUdR is a nucleoside analogue that is incorporated directly into DNA. Emission of AEs causes DNA DSBs resulting in chromosomal aberrations in DNA, causing profound cytotoxicity (Chan et al. 1976; Kassis et al., 1987). Chan et al. compared the clonogenic survival of V79 Chinese hamster (CHO) cells exposed *in vitro* to ^125^I-IUdR with ^3^H-deoxyuridine and ^131^I-iododeoxyuridine (^3^H-TdR; ^131^I-IUdR). ^125^I-IUdR yielded a steeply declining clonal survival curve with a small shoulder, typical of high-LET radiation, and ^125^I-IUdR was more cytotoxic than ^3^H-TdR or ^131^I-IUdR, which emit low energy or intermediate energy β-particles, respectively. Incubation of the cells for one hour with only 0.0037 Bq of ^125^I-IUdR generated 3 DNA DSBs per cell, but a 10-fold greater amount of ^131^I-IUdR and 17-fold higher amount of ^3^H-TdR were required for the same DNA damaging effect. The radiotoxicity of DNA-incorporating ^125^I-IUdR has also been shown in preclinical *in vivo* studies. Sahu et al. demonstrated that ^125^I-IUdR treatment prevented paralysis caused by leptomeningeal gliosarcoma metastases in rats (Sahu et al. 1997). A single intrathecal administration of 18.5 MBq of ^125^IUdR, given over 5 daily fractions or by continuous 5-day infusion prolonged time-to-paralysis to 11, 12 and 15 days respectively, from 9 days for control saline-treated mice. These early demonstrations of DNA-targeted AE radiotherapy in mammalian cell lines and aggressive preclinical animal tumour models illustrate the high cytotoxicity of AEs when emitted close to DNA.

More recently, a novel chemoradiotherapy strategy has been explored using AE-emitters. The chemotherapy drug cisplatin [*cis*-diaminedichloroplatinum (II)] forms DNA-platinum adducts in cells. This DNA crosslinking agent has been combined with radiotherapy, termed chemoradiotherapy, in the treatment of several solid cancers (Shrivastava et al. 2018; Psyrri et al. 2004; Zatloukal et al. 2004). Cisplatin and ionising radiation show synergism in their cell killing effects (Gorodetsky et al. 1998). Thus, combining cisplatin with radiotherapy using radionuclides of platinum that emit AEs (e.g. ^195m^Pt and ^191^Pt) is a rational approach. ^195m^Pt decay results in a larger number of AEs and a high energy per decay compared to other commonly used AE emitters such as ^125^I and ^111^In (Table 1). ^195m^Pt produced from irradiation of ^197^Au in a high current linear accelerator [^197^Au(γ,np)^195m^Pt] was extracted and used to synthesise ^195m^Pt-cisplatin with a high specific activity of up to 3.7 TBq/mg by Bodnar and colleagues (Dykiy et al. 2007; Bodnar et al. 2015). Treatment of Ehrlich adenocarcinoma cells *in vitro* with 0.017 pg/mL ^195m^Pt-cisplatin reduced cell viability to 3% within 6 h, an approximate 8-fold reduction compared to 7.5 μg/mL treatment with nonradioactive cisplatin, which decreased cell viability to only 25% (Bodnar et al. 2015). In vivo, male mice with subcutaneous Ehrlich adenocarcinoma xenografts in the right thigh received 5 intraperitoneal (i.p.) injections of normal saline (control) or 0.7 mg nonradioactive cisplatin on alternating days, or received a single injection of 0.017 pg ^195m^Pt (Bodnar et al. 2015). Mice were monitored for body weight and tumour size for 21 days. The single treatment with ^195m^Pt-cisplatin resulted in a 65% tumour growth inhibition, compared to 35% tumour growth inhibition with 5 treatments of nonradioactive cisplatin. While both nonradioactive and ^195m^Pt-cisplatin treatments resulted in significant decreases in body weight compared to the normal saline treated control mice, there were no significant differences in the body weights between radioactive and nonradioactive cisplatin treatments. These promising results suggest that combining an AE emitter with cisplatin chemotherapy greatly enhances the therapeutic effect of cisplatin on tumours while not increasing normal tissue toxicity.

Another platinum isotope, ^191^Pt has been investigated for chemoradiotherapy. ^191^Pt decays with a half-life of 2.8 days by EC, producing 17.8 KeV of AEs per decay (and 273 KeV/decay in γ-photons) (Table 1**,** Eckerman and Endo 2008). Areberg et al. produced ^191^Pt by proton irradiation (75–65 MeV) of gold foil [^197^Au(p,2p,5n)^191^Pt, ^197^Au(p,p6n)^191^Au➔^191^Pt, ^197^Au(p,7n)^191^Hg➔^191^Au➔^191^Pt] and synthesised ^191^Pt-cisplatin (Areberg et al. 1999). The *in vitro* cytotoxicity of ^191^Pt-cisplatin was assessed at increasing specific activities of 0–167 MBq/mg (0–20 μg/mL) on ME180 human cervical carcinoma cells to determine the IC_50_ values (Areberg et al. 2000) which is the concentration required to inhibit cell growth by 50%. The IC_50_ was reduced from 3.2 μg/mL for nonradioactive cisplatin, to 2.8 and 0.8 μg/mL for the lowest (48 MBq/mg) and highest (167 MBq/mg) specific activities, respectively. This resulted in enhancement ratios of 1.2 to 4.3 for ^191^Pt-cisplatin compared to nonradioactive cisplatin. The enhanced potency of ^191^Pt-cisplatin was subsequently shown in tumour-bearing BALB/c nude mice (Areberg et al. 2001). A cisplatin-sensitive patient-derived squamous cell carcinoma of the nasal cavity was subcutaneously inoculated into male and female mice. Treatment groups consisted of mice receiving i.p. normal saline, 5 mg/kg nonradioactive cisplatin administered i.p., or a single i.p. injection of 80 MBq/mg or 160 MBq/mg of ^191^Pt-cisplatin. Specific tumour growth delay (SGD, the normalised difference in tumour doubling time compared to normal saline treated control mice) was 2.1 for nonradioactive cisplatin, 3.0 for 80 MBq/mg ^191^Pt-cisplatin, and 3.9 for 160 MBq/mg ^191^Pt-cisplatin. It was calculated that for the same tumour growth delay effect obtained with a 5 mg/kg dose of 80 or 160 MBq/mg ^191^Pt-cisplatin, the dose of nonradioactive cisplatin would need to be 9 and 10 mg/kg, respectively.

Other DNA-binding radiotherapeutic agents that emit AEs have been studied. Anthracyclines (e.g. doxorubicin and daunorubicin) are planar aromatic molecules that intercalate between base-pairs in DNA. Radiolabelled anthracyclines could cause DNA damage by AE emission in addition to the chemotoxicity of these drugs. Ickenstein et al. demonstrated *in vitro* the potent radiotoxicity of radioiodinated daunorubicin (Ickenstein et al. 2006). Exposure of SK-BR-3 human breast cancer cells to ^125^I-labelled daunorubicin (50 kBq/mL; 0.5 ng/mL) was more than 4-orders of magnitude more cytotoxic than unlabelled daunorubicin or doxorubicin, or stable ^127^I-iodinated daunorubicin, demonstrating the increased potency imparted on these anthracycline drugs by labelling with ^125^I. Imstepf et al. studied a doxorubicin derivative conjugated to a dipicolylamine chelator for radiolabelling with ^99m^Tc tricarbonyl complex (Imstepf et al. 2015). The ^99m^Tc-labelled doxorubicin produced a strong cytotoxic effect on radiosensitive murine melanoma B16F1 cells. After 36 h of incubation at the maximum concentration of ^99m^Tc studied (10 MBq/mL), about 80% of B16F1 cells were killed, but only 30% and 25% of A431 human squamous carcinoma cells and HeLa cells were killed, respectively. The reduced cytotoxicity of ^99m^Tc-doxorubicin in these cells may reflect differences in radiosensitivity to AEs. Nonetheless, incubation with ^99m^TcO_4_^−^ which was not accumulated in the nucleus and did not bind to DNA, only killed about 10% of the cells for all three cell types. ^99m^Tc is not ideal as an AE-emitter for cancer radiotherapy because it emits 4.4 AEs per decay but there is a low total AE energy released per decay (0.9 keV) and a low average energy per AE (0.2 keV) (Table 1). However, there is a higher energy IC electron with average energy of 13.8 keV and total energy released per decay of 15.2 keV.

Acridine and acridone derivatives are an additional class of DNA intercalators that have been studied as platforms for delivery of AE-emitting radionuclides. Desbois et al. screened a panel of acridine and acridone derivatives for cytotoxicity in several human and murine melanoma cell lines and identified 7-iodo-acridone, and 5-iodo-acridine as two compounds that demonstrated cytotoxicity *in vitro* on M4Beu human melanoma cells (Desbois et al. 2008). The biodistribution of these compounds was studied in mice bearing B16F0 murine melanomas. Both ^125^I-labelled compounds accumulated in B16F0 tumours at 72 h post-injection, but 7-^125^I-iodo-acridone exhibited 2-fold greater uptake (17.5 vs. 6.9%ID/g). Gardette et al. investigated ^125^I-labelled acridine (^125^I-ICF01040) and acridone (^125^I-ICF01035) derivatives in both melanotic and amelanotic melanoma cells (Gardette et al. 2014). ^125^I-ICF01035 was concentrated mainly in melanosomes of B16F0 murine melanoma cells, but in A375 amelanotic human melanoma cells, ^125^I-ICF01035 was taken up into the nucleus. Despite these differences in subcellular distribution, ^125^I-ICF01035 was similarly cytotoxic to both cell types (50% growth inhibitory activity, A_50_ = 10–12 kBq/mL). ^125^I-ICF01040 which mainly accumulated in the cytoplasm, had significantly greater cytotoxicity in all cell lines and was the most cytotoxic in B16F0 cells (A_50_ = 2 kBq/mL). This study demonstrated that although nuclear localisation resulted in the greatest cytotoxicity, cytoplasmic distribution also resulted in killing of melanoma cells *in vitro*.

### Targeted Radiotherapeutic agents

The epidermal growth factor receptor (EGFR) is the first member the EGFR family which also includes HER2, HER3 and HER4 (Olayioye et al. 2000). EGFR overexpression is a hallmark of many epithelial-derived cancers (Salomon et al. 1995). Michel et al. reported killing of EGFR-positive vulvar squamous cell carcinoma A431 cells *in vitro* by ^111^In- or ^125^I-labelled anti-EGFR antibodies (Michel et al. 2004). At the highest concentration studied (1.5 MBq/mL), 93–100% of these cells were killed. ^131^I-anti-EGFR antibodies also killed A431 cells, but these effects were not targeted, and mostly resulted from β-particle emissions in the growth medium (i.e. cross-fire effect). As discussed earlier, nuclear importation is an important property that maximises the lethal DNA damaging effects of AEs. Our group modified ^111^In-labelled anti-EGFR mAb nimotuzumab with 13-mer peptides CGYG*PKKKRKV*GG that harbour the SV-40 large T-antigen-derived nuclear localisation sequence (NLS; italicised) and studied the cytotoxicity of these radioimmunoconjugates towards EGFR-positive breast cancer cells (Fasih et al. 2012). The NLS peptides are recognised by importins α/β that function to shuttle cytoplasmic proteins across the nuclear pore complex (Costantini et al. 2008b). NLS peptide modification enhanced nuclear uptake 2-fold in EGFR-positive MDA-MB-468 human breast cancer cells compared to ^111^In-nimotuzumab without NLS. However, ^111^In-nimotuzumab without NLS still accumulated in the nucleus, probably mediated by an endogenous NLS in the transmembrane domain of the EGFR (Wang and Hung 2012). Nonetheless, the incorporation of NLS rendered ^111^In-NLS-nimotuzumab 7-fold more potent in reducing the CS of MDA-MB-468 cells and caused 2-fold more DNA DSBs detected by immunofluorescence for γH2AX foci in the nucleus (Fasih et al. 2012).

^111^In-DTPA-hEGF is an AE-emitting radiotherapeutic agent for EGFR-positive breast cancer previously studied by our group (Reilly et al. 2000).^111^In-DTPA-hEGF exhibited rapid receptor-mediated binding and internalisation into MDA-MB-468 cells and importation into the cell nucleus within 24 h, likely mediated by the endogenous NLS in the EGFR. MDA-MB-468 cells exposed to ^111^In-DTPA-hEGF exhibited decreased CS which was directly correlated with increased density of DNA DSBs in the cell nucleus assessed by immunofluorescence for γH2AX (Cai et al. 2008). ^111^In-DTPA-hEGF administered i.v. in fractionated doses over 5 weeks (total 28–92 MBq; 5–17 μg) significantly inhibited the growth of MDA-MB-468 tumours in mice, and was particularly effective for treatment of small tumour xenografts (Chen et al. 2003). Some decreases in white blood cell (WBC) and platelet counts were observed, but these remained within the normal range. There was no hepatic or renal toxicity. Subsequent preclinical translational bridge studies revealed that administered amounts of ^111^In-DTPA-hEGF up to 44 MBq (3–30 μg) in mice or 85.1 MBq (58 μg) in rabbits, corresponding to 44-times and 1-times the maximum planned human administered amount of radioactivity for a Phase 1 clinical trial were safe (Reilly et al. 2004). The projected whole-body effective dose in humans was 0.19 mSv/MBq and the projected doses to the liver and kidneys were within safe limits for the Phase 1 trial (see Clinical Studies section). Despite these encouraging results, bioactive peptides such as hEGF may cause adverse effects in humans when used for radiotherapy. In the subsequent Phase 1 trial of ^111^In-DTPA-hEGF, patients experienced flushing, hypotension and nausea, which was attributed to the hEGF moiety (see Clinical Studies section) (Vallis et al. 2014). We studied an ^111^In-labelled truncated form of hEGF (^111^In-EGFt) that exhibits decreased biological activity (Panosa et al. 2015). ^111^In-DTPA-EGFt bound with lower affinity to the EGFR than ^111^In-DTPA-hEGF (K_d_ = 6.0 × 10^− 8^ M vs. 1.3 × 10^− 9^ M, respectively) but nevertheless was internalised and imported into the nucleus of MDA-MB-468 cells. However, ^111^In-DTPA-EGFt was 2–8 fold less cytotoxic to MDA-MB-468 cells *in vitro* than ^111^In-DTPA-hEGF and exhibited 2.2-fold lower tumour uptake *in vivo* in mice with MDA-MB-468 xenografts.

**Fig. 7:**
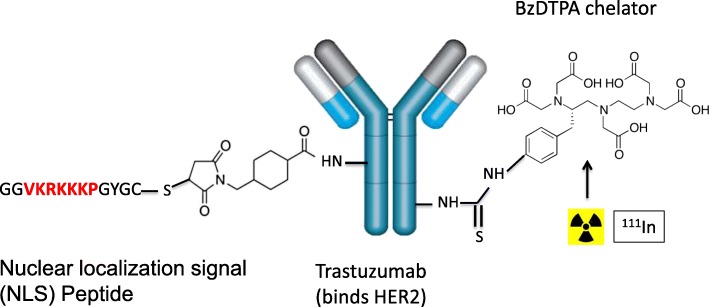
^111^In-NLS-trastuzumab is an example of a targeted Auger electron (AE)-emitting radioimmunotherapeutic (RIT) agent composed of the anti-HER2 monoclonal antibody, trastuzumab modified with nuclear translocation sequence (NLS in red) peptides and benzylisothiocyanate DTPA (BzDTPA) to complex ^111^In. ^111^In-NLS-trastuzumab is internalised by HER2-overexpressing breast cancer cells and is transported to the cell nucleus, where the AEs cause lethal DNA double-strand breaks (DSBs) (Costantini et al. 2007)

**Fig. 8:**
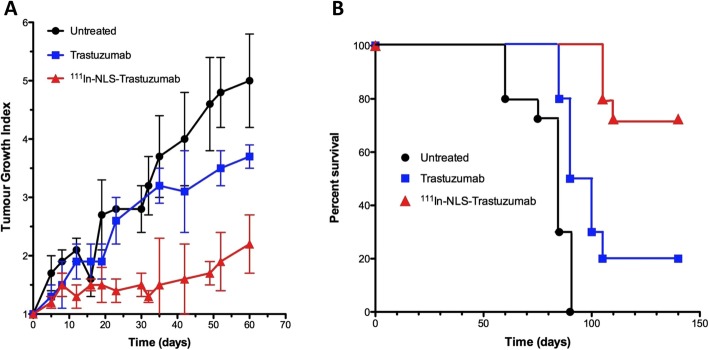
**a** Treatment of athymic mice with subcutaneous HER2-positive MDA-MB-361 human breast cancer xenografts with a single injection of ^111^In-NLS-trastuzumab (9.25 MBq; 4 mg/kg) significantly slowed tumour growth compared to control mice receiving unlabelled trastuzumab (4 mg/kg) or normal saline. **b** Treatment of tumour-bearing mice with two injections of ^111^In-NLS-trastuzumab (9.25 MBq; 4 mg/kg) significantly prolonged survival compared to control mice receiving unlabelled trastuzumab or normal saline (Costantini et al. 2010)

HER2 is the second member of the EGFR family. HER2 is overexpressed on 15–25% of breast cancers and is the target for treatment with trastuzumab (Herceptin; Roche), pertuzumab (Perjeta; Roche) and trastuzumab-emtansine (Kadcycla; Roche) (Larionov 2018). We modified trastuzumab with NLS peptides and derivatised these immunoconjugates with DTPA to complex ^111^In for AE RIT of HER2-positive breast cancer (Costantini et al. 2007) (Fig. 7). ^111^In-DTPA-NLS-trastuzumab was bound and internalised by HER2-positive breast cancer cells and transported to the nucleus. Exposure of SK-BR-3 human BC cells *in vitro* to ^111^In-DTPA-NLS-trastuzumab (70 μg/mL; 240 MBq/mg) caused extensive DNA DSBs, reducing their clonogenic survival by > 90% (Costantini et al. 2007). In contrast, unlabelled trastuzumab (70 μg/mL) decreased the survival of SK-BR-3 cells by only 35%. ^111^In-DTPA-NLS-trastuzumab exhibited high tumour uptake (12.1%ID/g) at 72 h p.i. in mice with HER2-overexpressing MDA-MB-361 human breast cancer xenografts (Costantini et al. 2007). For RIT studies, an administered radioactivity amount level of ^111^In-DTPA-NLS-trastuzumab that caused no observable adverse effects (NOAEL) on hematopoietic, liver or kidney function and on body weight was first determined (Costantini et al. 2010). A single injection at the NOAEL (9.25 MBq; 4 mg/kg) administered to mice with subcutaneous MDA-MB-361 xenografts significantly inhibited tumour growth by 4-fold (Costantini et al. 2010) (Fig. 8a). An equivalent single mass dose (4 mg/kg) of unlabelled trastuzumab was 3-fold less effective than ^111^In-DTPA-NLS-trastuzumab. Treatment of mice with MDA-MB-361 tumours with two injections of ^111^In-DTPA-NLS-trastuzumab (9.25 MBq; 4 mg/kg each) separated by two weeks significantly prolonged survival compared to mice treated with unlabelled trastuzumab or untreated mice (Fig. 8b).

Our group extended this approach to RIT of acute myeloid leukemia (AML) using ^111^In-labelled anti-CD33 murine M195 and humanised HuM195 mAbs (Chen et al. 2006) similarly modified with NLS peptides to promote nuclear importation. ^111^In-NLS-HuM195 exhibited 2-fold greater potency than ^111^In-HuM195 without NLS for killing HL-60 human myeloid leukemia cells *in vitro*, and also decreased the clonogenic survival of CD33-positive primary patient AML specimens (Chen et al. 2006). Moreover, ^111^In-NLS-HuM195 retained its cytotoxicity *in vitro* against HL-60-MX-1 cells, which are a mitoxantrone-resistant subclone of HL-60 cells as well as on primary AML specimens that expressed multidrug resistance (MDR) transporters (Kersemans et al. 2008). These results suggest that RIT with AEs could be a promising approach to treatment of AML and may be able to overcome chemotherapy resistance, which is a challenge in treating this disease. Our subsequent studies focused on anti-CD123 (interleukin-3 receptor, IL-3, α-subchain) murine 7G3 or humanised CSL360 mAbs (CSL Ltd., Parkville, Australia) modified with NLS and labelled with ^111^In (Leyton et al. 2011). Expression of CD123 in the absence of CD131 (IL-3R β-subchain) is a phenotype that is expressed by leukemic stem cells (LSCs). LSCs are believed to cause AML and their survival after treatment is implicated in recurrence (Jordan et al. 2000). ^111^In-NLS-7G3 and ^111^In-NLS-CSL360 killed AML-5 human myeloid leukemia cells expressing the CD123^+^/CD131^−^ phenotype of LSCs (Gao et al. 2016; Leyton et al. 2011). Combining ^111^In-NLS-CSL360 with inhibitors of DNA repair increased their cytotoxicity *in vitro* (Zereshkian et al. 2014). These RICs localised in the bone marrow (BM) and spleen and at other extramedullary sites of leukemia in NOD/SCID mice engrafted with AML-5 cells or primary AML specimens, detected by microSPECT/CT (Leyton et al. 2011, 2014). However, we encountered two challenges in studying RIT of AML with ^111^In-NLS-CSL360 preclinically in AML-engrafted mice. The mouse model which was used to identify LSCs and is also used to study new treatments for leukemia is the NOD/SCID mouse engrafted into the BM with primary AML cells (Bonnet et al. 1999). However, NOD/SCID mice harbour a germ-line mutation in DNA repair, which renders these mice unusually sensitive to radiation, including to RIT, and limits the amount of radioactivity which may be safely administered. Moreover, NOD/SCID mice require pre-treatment with 200 cGy of X-radiation to enable AML engraftment. We found that paradoxically, RIT with ^111^In-NLS-CSL360 encouraged AML engraftment rather than decreased engraftment, due to a boost in radiation to the BM caused by the γ-photons from ^111^In which further primed the BM niche for leukemic cell engraftment (Bergstrom et al. 2016). NRG (NOD-*Rag1*^*null*^ *IL2rg*^*null*^, NOD rag gamma) mice that do not harbour this DNA repair defect may be used to study RIT, but these mice are more immunocompromised than NOD/SCID mice and AML engraftment efficiency is often very high, resulting in a high tumour burden that is difficult to eradicate. Further refinements to the mouse model of AML are required to study AE-mediated RIT of AML. Other researchers in the AE field have also previously studied ^111^In- or ^67^Ga-labelled mAbs recognising MHC-II antigens or CD20 for RIT of B-cell lymphomas with promising results *in vitro* (Michel et al. 2003) as well as *in vivo* (Michel et al. 2005).

Somatostatin receptor subtype-2 (sst-2) which is overexpressed on neuroendocrine malignancies is another attractive target for radiotherapy with AEs. Octreotide is a stable octapeptide analog of somatostatin that preferentially binds with high affinity to sst-2. Capello et al. studied ^111^In-DTPA-octreotide for peptide receptor radionuclide therapy (PRRT) exploiting the AE emissions of ^111^In (Capello et al. 2003). Exposure of sst-2 positive CA20948 rat pancreatic tumour cells *in vitro* to ^111^In-DTPA-octreotide reduced their survival to 0%, while non-targeted ^111^In-DTPA had no inhibitory effects and unlabelled octreotide (1 μM) reduced survival by only 40%. In a subsequent *in vivo* study, Capello et al. showed that treatment of rats with CA20948 tumours with a single injection of 370 MBq or 3 injections of 111 MBq each of ^111^In-DTPA-octreotide yielded complete responses in small tumours and partial responses in larger tumours (Capello et al. 2005). ^111^In-DTPA-Octreotide has also been examined clinically for AE radiotherapy in patients with neuroendocrine malignancies (see Clinical Studies section).

Prostate-specific membrane antigen (PSMA) is a cell surface glycoprotein overexpressed on prostate cancer, but only found at low levels in other tissues such as the salivary gland, proximal small intestine and kidneys (Silver et al. 1997; Ghosh and Heston 2004). PSMA is therefore an attractive target for AE radiotherapy of prostate cancer. ^161^Tb is a radionuclide with a half-life of 6.89 days that emits a total of 5.1 keV of AE energy per decay with an average AE energy of 5.7 keV (Table 1). ^161^Tb also emits γ-photons at 49 keV and 75 keV suitable for SPECT imaging (Uusijärvi et al. 2006) and thus may have theranostic application for imaging and treatment of cancer. ^161^Tb-PSMA-617 was more potent for killing PSMA-positive PC-3 PIP tumour cells than PSMA-617 labelled with the β-emitter, ^177^Lu (^177^Lu-PSMA-617) when these cells were exposed *in vitro* to 0.05–10 MBq/mL (Müller et al. 2019). There was no effect on the viability of PSMA-negative PC-3 flu cells up to concentrations of 10 MBq/mL. In PC-3 PIP tumour-bearing athymic mice, ^161^Tb-PSMA-617 treatment achieved tumour growth delay, which was dependent on the amount administered. An administered amount of 10.0 MBq prolonged the time for tumour size to reach the end-point to 42 days vs. 28 days for 5.0 MBq and prolonged survival (65 days vs. 36 days, respectively). Tumour-inhibitory effects were more pronounced in mice treated with ^161^Tb-PSMA-617 than ^177^Lu-PSMA-617 when administered at 2.5 MBq, 5.0 MBq and 10.0 MBq.

### Overcoming receptor heterogeneity in AE radiotherapy

A strength of AEs for cancer therapy is that their cytotoxicity is restricted mostly to cells that bind and internalise the targeting agent. There is no cross-fire effect but only a local cross-dose effect, which provides a high specificity for killing cancer cells. However, this poses a challenge since heterogeneity in target expression may allow some cells that are target-negative to escape the lethal effects of the AEs. In contrast longer range β-emitters, such as ^177^Lu and ^90^Y, have a cross-fire effect that is able to kill non-targeted cells within the range of the β-particles. The bystander effect of AEs may help to overcome the obstacle of receptor heterogeneity as AE-emitters from targeted irradiated cancer cells may still exert cytotoxic effects on non-targeted cells. The bystander effect describes the biological effects of radiation on cells that have not been directly irradiated (Marín et al. 2015). One of the first demonstrations of the bystander effect from AEs was reported by Xue et al. in nude mice inoculated with a mixture of untreated LS174T human colon cancer cells and cells loaded with lethal amounts of ^125^I-IUdR (Xue et al. 2002). Tumour growth was reduced significantly compared to inoculation of only untreated cells, yet the range of the AEs from ^125^I was subcellular (< 0.5 μm) and the absorbed dose deposited in the untreated cells from ^125^I-IUdR incorporated into the DNA of treated cells was < 10 cGy. The effects on tumour growth caused by the ^125^I-IUdR was therefore interpreted as a bystander effect. In another study, UVW human glioma cells and EJ138 human bladder carcinoma cells transfected with the noradrenaline transporter (NAT) gene that were exposed to medium from cells that accumulated ^123^I-MIBG showed a decrease in survival, indicating that mediators of the bystander effect were released into the medium from irradiated cells (Boyd et al. 2006).

Another strategy to address receptor heterogeneity is to design radioimmunoconjugates that recognise more than one receptor. We synthesised bispecific radioimmunoconjugates (bsRICs) that bind HER2 and EGFR (Razumienko et al. 2013, 2016) since upregulation of the EGFR and co-expression of EGFR and HER2 is a frequent mechanism of resistance to HER2-targeted therapies (Gallardo et al. 2012). These bsRICs were constructed by conjugating trastuzumab Fab fragments which bind HER2 through a flexible 24-mer PEG (PEG_24_) spacer to human EGF (hEGF). The immunoconjugates were modified with DTPA for labelling with ^111^In or DOTA for complex ^177^Lu (Razumienko et al. 2016). We also designed analogous bsRICs labelled with ^64^Cu for PET of tumours that co-express HER2 and EGFR (Kwon et al. 2017). In clonogenic assays, monospecific ^177^Lu- and ^111^In-trastuzumab Fab or EGF only killed tumour cells that expressed HER2 or EGFR, respectively, while the bsRICs were able to kill cells that expressed HER2 or EGFR or both receptors. These bsRICs were also more potent than the monospecific agents. ^111^In-labelled bsRICs were less effective than ^177^Lu-labelled bsRICs for treatment of MDA-MB-231/H2N tumours in mice that co-express HER2 and EGFR, but in this study, the same amount of radioactivity was administered for both bsRICs (11.1 MBq; 10 μg). This administered amount caused no observable normal tissue toxicity (NOAEL), which suggests that higher amounts of the ^111^In-labelled bsRICs could be safely administered to increase the therapeutic effects. It has been previously shown that RIT with AE-emitters yields more potent tumour growth-inhibitory effects in mice than β-emitters when administered at equitoxic but not necessarily equal amounts of radioactivity (Behr et al. 2000). Both ^111^In- and ^177^Lu-labelled bsRICs were also effective for treatment of TrR1 human breast cancer xenografts that are HER2 and EGFR-positive, but have acquired resistance to trastuzumab, but these tumours were less sensitive than MDA-MB-231/H2N tumour xenografts (Razumienko et al. 2016).

Modular nanotransporters (MNTs) represent an interesting and unique platform for targeting and internalising AE-emitters into cancer cells and delivering these radionuclides to the cell nucleus (Sobolev 2018). MNTs are recombinant multifunctional polypeptides that combine modules for receptor binding, internalisation, endosomal escape and nuclear importation. Slastnikova et al. reported that ^125^I-labelled EGFR-targeted MNTs (^125^I-N-succinimidyl-4-guanidinomethyl-3-[^125^I] iodobenzoate (SGMIB)-MNT) efficiently transported over 60% of the internalised radioactivity into the nucleus of EGFR-positive human epidermoid carcinoma A431 cells and human glioblastoma D247 MG cells after 1 h of incubation *in vitro* (Slastnikova et al. 2012). The cytotoxicity of the ^125^I-MNTs was dependent on EGFR density with greater killing observed for EGFR-overexpressing A431 cells than D247 cells with low EGFR expression.

### Requirement for nuclear localisation of AE-emitters

Nuclear translocation may not be an absolute requirement for the cytotoxic effects of AE-emitters. Using ^125^I-labelled non-internalizing anti-CEA murine IgG1K 35A7 mAb, internalizing anti-HER2 trastuzumab mAb or anti-EGFR hybridoma murine m225 mAb and Tat cell penetrating peptide targeting the cell membrane, cytoplasm or the cell nucleus, respectively, Pouget et al. demonstrated that decreases in clonogenic survival of A-431 human vulvar squamous carcinoma and SKOV-3 human ovarian cancer cells were significantly higher for cell membrane than for cytoplasmic localisation (Pouget et al. 2008). Therefore, non-internalizing cell surface biomarkers such as carcinoembryonic antigen (CEA) may represent feasible targets for AE radiotherapies. Santoro et al. showed that ^125^I-35A7 anti-CEA mAbs significantly increased survival in nude mice bearing intraperitoneal EGFR-positive vulvar squamous cell carcinoma xenografts transfected to express CEA compared to treatment with unlabelled mAbs (59 days vs. 24 days) (Santoro et al. 2009). The effect was drastically reduced for ^125^I-labelled internalizing anti-EGFR mAbs and the corresponding unlabelled mAbs (77 days vs 76 days, respectively), however, this lower effectiveness could be explained by the catabolism of the internalised ^125^I-labelled mAbs with export of released ^125^I from the tumour cells (Santoro et al. 2009). Interestingly, Piron et al. showed the accumulation of unrepaired DNA DSBs over time after exposure to either ^125^I-labelled non-internalizing anti-CEA mAbs or internalizing anti-EGFR mAbs in human colorectal cancer HCT-116 cells (Piron et al. 2014). There was a lack of linear dose-effect relationship between the biological response observed and the absorbed dose deposited by the anti-CEA and anti-EGFR ^125^I-labelled mAbs, suggesting ^125^I may cause bystander effects on the cell-membrane (Piron et al. 2014).

### Radiation nanomedicines

Nanoparticles labelled with AE-emitting radionuclides have also been studied for cancer treatment. Fonge et al. reported the construction of ^111^In-labelled BCMs modified with hEGF to target EGFR-positive breast cancer cells (Fonge et al. 2009). These ^111^In-hEGF-BCMs showed EGFR density-dependent cellular uptake and nuclear importation in a panel of human breast cancer cell lines (MDA-MB-468, MDA-MB-231 and MCF-7). Exposure of MDA-MB-468 cells with high EGFR expression to ^111^In-hEGF-BCMs reduced the clonogenic survival of these cells to 2.6%, but treatment of these cells with ^111^In-DTPA-hEGF was more effective, reducing the survival to 0.4%. Non-targeted ^111^In-BCMs were not effective for killing MDA-MB-468 cells, and ^111^In-hEGF-BCMs did not kill MDA-MB-231 or MCF-7 cells with intermediate or low EGFR expression, respectively. Hoang et al. reported the synthesis of BCMs incorporating polymers with DTPA to complex ^111^In, Fab fragments to bind HER2-positive breast cancer cells and NLS peptides to enable nuclear localisation (Hoang et al. 2012). The BCMs also incorporated methotrexate as a radiosensitiser, since this was previously shown to enhance the cytotoxicity of ^111^In-NLS-trastuzumab (Costantini et al. 2008a). Uptake of ^111^In-trastuzumab-Fab-BCMs in human breast cancer cells was dependent on the level of HER2 expression, and incorporation of NLS peptides enhanced the nuclear uptake of ^111^In. The clonogenic survival of SK-BR-3 cells, MDA-MB-361 and MDA-MB-231 cells with high, intermediate or low HER2 expression were reduced to approximately 23%, 45% and 77%, respectively, after exposure to ^111^In-NLS-trastuzumab-BCMs for 24 h *in vitro*.

**Fig. 9:**
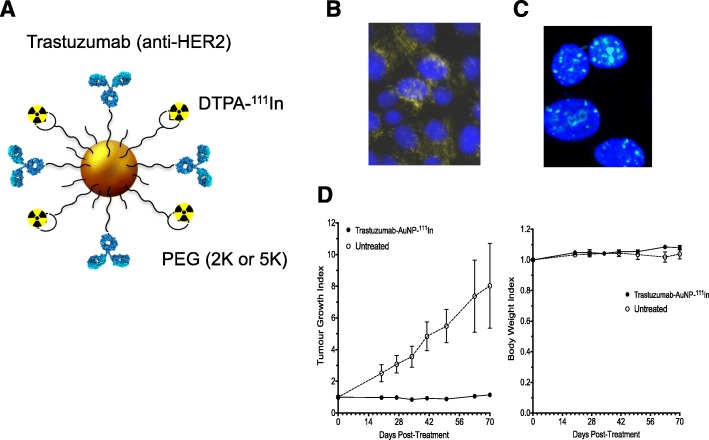
**a** ^111^In-trastuzumab-AuNPs are an example of an Auger electron (AE)-emitting radiation nanomedicine composed of gold nanoparticles (AuNPs; 30 nm) modified with 2 kDa polyethylene glycol (PEG) chains to stabilise the AuNPs and longer 5 kDa PEG chains conjugated to trastuzumab to bind HER2 or to DTPA to complex ^111^In. **b** Dark-field and fluorescence microscopy demonstrating peri-nuclear localisation (nucleus is stained blue with DAPI) of ^111^In-trastuzumab-AuNPs (yellow) in HER2-positive SK-BR-3 human breast cancer cells likely mediated by an endogenous nuclear localisation sequence (NLS) peptide in HER2. **c** DNA double-strand breaks (DSBs; bright foci) detected by immunofluorescence for γH2AX in the nucleus of SK-BR-3 cells exposed to ^111^In-trastuzumab-AuNPs mediated by emission of AEs by ^111^In. **d** Local intratumoural (i.t.) injection of ^111^In-trastuzumab-AuNPs (10 MBq) in athymic mice with subcutaneous HER2-positive MDA-MB-361 human breast cancer xenografts arrested tumour growth compared to untreated mice (left panel) with no change in body weight (right panel) indicating no generalised normal tissue toxicity (Cai et al. 2016)

Gold nanoparticles (AuNPs) have also been studied for AE radiotherapy. Song et al. reported the synthesis of ^111^In-labelled EGF-conjugated AuNPs (^111^In-EGF-AuNPs) (Song et al. 2016). Binding and internalisation of ^111^In-EGF-AuNPs by breast cancer cells was EGFR-dependent and was 12-fold higher for MDA-MB-468 than MCF-7 human breast cancer cells with high or low EGFR expression, respectively. Exposure of MDA-MB-468 cells for 4 h to ^111^In-EGF-AuNPs reduced the clonogenic survival to 17%, while less than 10% decreased survival was found for MCF-7 cells. A major limitation to systemic (i.v.) administration of radiolabelled AuNPs is high liver and spleen uptake mediated by interactions with the mononuclear phagocyte system (MPS), resulting in low tumour uptake (1–2%ID/g) in mouse tumour xenograft models (Chattopadhyay et al. 2012). Surface coating of nanoparticles with polyethylene glycol (PEG) can reduce MPS recognition. Song et al. constructed ^111^In-labelled PEGylated AuNPs modified with EGF to target EGFR for AE radiotherapy (Song et al. 2017). In mice with EGFR-positive MDA-MB-468 xenografts, liver uptake of i.v. injected of ^111^In-EGF-PEG-AuNPs was 3-fold lower than non-PEGylated ^111^In-EGF-AuNPs. Co-administration of an excess (15 μg) of EGF to block uptake by EGFR on hepatocytes improved tumour uptake from 2.8%ID/g to 3.9%ID/g. An alternative to systemic administration of radiolabelled AuNPs for cancer treatment is intratumoural (i.t.) injection, since this greatly minimises liver and spleen uptake due to retention of the AuNPs at the local injection site. We synthesised trastuzumab-modified AuNPs labelled with ^111^In for local treatment of HER2-positive breast cancer (Cai et al. 2016) (Fig. 9a). These ^111^In-trastuzumab-AuNPs were bound by HER2-positive SK-BR-3 or MDA-MB-361 human breast cancer cells and were internalised to a peri-nuclear location, likely mediated by a nuclear translocation sequence (NLS) in HER2 (Chen et al. 2005) (Fig. 9b). The emission of AEs by ^111^In caused lethal DNA DSBs in SK-BR-3 cells (Fig. 9c) reducing their CS by 3-fold. A single i.t. injection of ^111^In-AuNPs (10 MBq; 2.6 × 10^12^ AuNPs) arrested the growth of s.c. MDA-MB-361 tumours in mice without normal tissue toxicity (Fig. 9d). These results are promising for local injection of AuNPs labelled with AE-emitters for tumours that are accessible.

## Clinical studies

There have been only a few clinical studies of AEs for cancer therapy and some of these trials were conducted 20 years ago. Nonetheless, it is helpful to review the results of these trials to appreciate the potential clinical feasibility of AEs for cancer treatment. Macapinlac et al. conducted a pilot clinical trial of co-administration of ^125^I-IUdR (185 MBq) or ^131^I-IUdR (370 MBq) to evaluate dosimetry and safety in 4 patients with colorectal cancer metastatic to the liver using hepatic artery infusion (Macapinlac et al. 1996). No tumour responses were noted or expected at these low amounts of administered radioactivity. Images revealed no retention in the BM or in other normal tissues. No side effects or hematologic toxicity were observed. Tumour DNA samples showed higher incorporation of radioactivity compared to normal hepatocyte DNA. This study suggests that hepatic artery infusion may be a feasible route of delivery of AE radiopharmaceuticals for treatment of hepatic metastases, although studies at therapeutic doses would be required to assess the effectiveness of this approach. Rebischung et al. performed intrathecal injection of ^125^I-IUdR in a patient with advanced pancreatic cancer with resistant neoplastic meningitis (Rebischung et al. 2008). The patient was given 4 doses of methotrexate prior to and after administration of 1850 MBq of ^125^I-IUdR. The treatment yielded clinical improvement correlated with a dramatic decrease in cerebrospinal carbohydrate antigen 19.9 (CA19.9) from 202 U/mL on Day 0 to 9 U/mL on Day 26. No central nervous system toxicity was found. Unfortunately, the disease recurred and the patient ultimately died.

Krenning et al. reported a Phase 1 clinical trial of ^111^In-DTPA-octreotide in 30 patients with advanced sst-2 positive neuroendocrine malignancies (Krenning et al. 1999). Up to 14 doses of ^111^In-DTPA-octreotide at 6 to 7 GBq each were administered with at least a two week interval and a maximum cumulative radioactivity amount of 74 GBq. Among the 21 patients who received a total of > 20 GBq, 8 patients showed stable disease (SD) and 6 patients demonstrated a reduction in tumour size. No major side effects were noted up to 2 years post-treatment except for a transient drop in platelets and lymphocytes in some patients. Valkeman et al. reported a Phase 1 trial of ^111^In-DTPA-octreotide administered at intervals of 2 weeks to several months in 12 or more doses at 2 to 11 GBq to a total of 20–160 GBq in 50 patients with sst-2-positive tumours (Valkema et al. 2002). Among 40 evaluable patients, therapeutic benefit was achieved in 21 patients (52.5%) with stable disease in 14 patients, minor remissions in 6 patients and a partial remission in 1 patient. Mild hematopoietic toxicity was found in most patients, but myelodysplastic syndrome or leukemia developed in 3/6 patients who received a total dose > 100 GBq. Impairment in spermatogenesis was indicated in male patients who showed a decrease in serum inhibin B and concomitant increase of serum FSH. The radiation dose deposited in the kidneys from ^111^In-DTPA-octreotide was 0.45 mGy/MBq, corresponding to a total of 45 Gy for a total administered radioactivity of 100 GBq, which is twice the accepted limit for external beam radiation. However, none of these patients developed the sequelae expected for renal toxicity such as hypertension, proteinuria or any changes in serum creatinine or creatinine clearance, suggesting that this organ dosimetry did not predict renal toxicity from ^111^In-DTPA-octreotide. As mentioned above (see Radiation Dosimetry section) the regional distribution of ^111^In-DTPA-octreotide in the kidneys mainly in the renal tubules combined with the very short range of the AEs emitted by ^111^In, may protect the more radiation sensitive glomeruli (Konijnenberg et al. 2004). Limouris et al. infused ^111^In-DTPA-octreotide (average administered amount 6.3 GBq) via the hepatic artery in 17 patients with inoperable sst-2-positive liver metastases (Limouris et al. 2008). One patient achieved a complete response, while 8 patients exhibited a partial remission and 3 patients had stable disease. The median survival among the 12 responding patients was 32 months. Mild (grade 1) erythrocytopenia, leukocytopenia and thrombocytopenia were found in 3 patients.

**Fig. 10:**
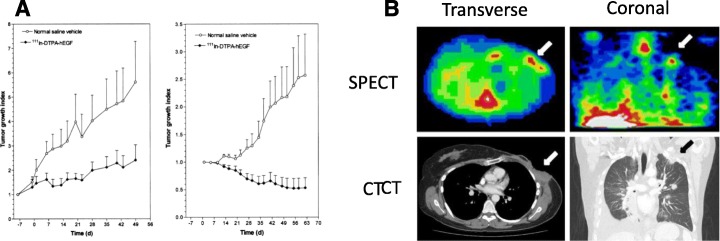
**a** Treatment of athymic mice with subcutaneous EGFR-positive MDA-MB-468 human breast cancer xenografts with 5 weekly amounts of ^111^In-DTPA-hEGF (cumulative dose, 92.5 MBq; 17 μg). Auger electron (AE) radiotherapy was effective compared to control mice treated with normal saline, but the growth of smaller, non-established tumours (right panel) was more strongly inhibited than larger tumours (left panel) (Chen et al. 2003). **b** SPECT and corresponding CT images of two patients at 24 h after injection of ^111^In-DTPA-hEGF in a Phase 1 clinical trial, demonstrating uptake into a recurrent primary breast cancer (left panels) or a lung metastasis (right panels) (Vallis et al. 2014)

Based on promising preclinical studies, our group conducted a Phase 1 clinical trial of ^111^In-DTPA-hEGF in 16 patients with metastatic EGFR-positive breast cancer administered 370–2220 MBq (0.25 mg) (Vallis et al. 2014) (Fig. 10). SPECT was used to assess the tumour and normal tissue uptake of ^111^In-DTPA-hEGF and to estimate radiation doses to normal organs. Toxicity was also evaluated. At these administered amounts, there were no hematopoietic, renal or hepatic toxicities. The estimated radiation dose to the whole body was 0.06 mGy/MBq, corresponding to 0.133 Gy at the maximum amount administered (2220 MBq). Following administration of 2200 MBq of ^111^In-DTPA-hEGF, the radiation dose to the kidneys (1.64 Gy) and liver (1.9 Gy) were within the radiation toxicity limit for these organs of 23 and 30 Gy, respectively, based on external beam radiotherapy (EBRT). However, some adverse effects were found that were related to the hEGF moiety. These included flushing, chills, nausea, and vomiting. One patient experienced grade 3 thrombocytopenia, but this was attributed to cancer metastasis to the BM rather than an adverse effect of ^111^In-DTPA-hEGF. No other patients experienced a serious adverse reaction. SPECT showed accumulation of ^111^In-DTPA-hEGF at known sites of breast cancer in 7/15 evaluable patients. Further dose-escalation of ^111^In-DTPA-hEGF is required to achieve a therapeutic effect, but the adverse effects associated with the hEGF moiety may require a higher SA than employed in this trial, in order to minimise the mass of hEGF injected. ^111^In-labelled anti-EGFR mAbs may be a promising alternative for AE-radiotherapy of EGFR-overexpressing breast cancer that would not cause these adverse effects (as discussed in the Preclinical Studies section). Our group is planning a Phase 1 clinical trial of ^111^In-NLS-trastuzumab to study its uptake in HER2-positive breast cancer by SPECT. Based on the results of this trial, we aim to conduct future therapeutic studies aimed at treatment of HER2-positive breast cancer with ^111^In-NLS-trastuzumab.

Li et al. reported a Phase 2 clinical trial of adjuvant RIT with ^125^I-labelled murine anti-EGFR mAb 425 in 192 patients with glioblastoma multiforme (GBM) (Li et al. 2010). Up to 3 weekly intravenous injections of ^125^I-mAb 425 (1.8 GBq each) were administered with a maximum cumulative radioactivity amount of 5.4 GBq. Among these 192 patients, 132 patients received RIT alone, and 60 patients received RIT and temozolomide (TMZ) chemotherapy. The median overall survival of 97 patients who received RIT alone was 14.5 months (range 12.1–16.7 months), and for 51 patients who received RIT and TMZ, the overall median survival was 20.4 months (range 14.9–25.8 months). Both treatment arms resulted in significantly improved overall survival compared to a historical control group of 39 patients receiving standard-of-care treatment, who had a median survival of 10.2 months (range 8.4–12.0 months). No grade 3 or 4 toxicities were observed for either of the RIT treatment groups, with only 7 of the 192 patients experiencing acute side effects (transient flushing, grade 1 nausea, hypotension and skin irritation). Four patients developed human anti-mouse antibodies (HAMA) preventing further administration of ^125^I-mAb 425, which was a murine mAb. The development of humanised or chimeric (e.g. cetuximab) and fully human anti-EGFR mAbs (e.g. panitumumab) in recent years should obviate this immunogenicity issue.

## Conclusions

AEs have very attractive properties for cancer therapy since their nanometre-micrometre range results in high LET that is potent for causing lethal damage in cancer cells. Biomolecules (mAbs and peptides), nucleosides and nanoparticles have been labelled with AE-emitting radionuclides (e.g. ^111^In, ^67^Ga, ^99m^Tc, ^123^I or ^125^I) and studied for cancer treatment. Several concepts have emerged. Firstly, AEs are especially lethal to cancer cells when emitted in close proximity to the cell nucleus, and particularly if the AE-emitting radiotherapeutic agent is incorporated directly into DNA (e.g. ^125^I-IUdR). Strategies that promote the delivery of AE-emitters to the nucleus by conjugating internalising mAbs to NLS peptides (e.g. ^111^In-NLS-trastuzumab), or targeting a receptor that harbours an endogenous NLS to enable nuclear uptake (e.g. EGFR or HER2) amplifies the lethal DNA-damaging effects of AEs. Nonetheless, nuclear localisation is not an absolute requirement and AEs can also kill targeted cancer cells by damaging the cell membrane, or non-targeted cells by a local cross-dose effect or a longer range bystander effect. Numerous studies have shown that AEs can kill cancer cells *in vitro* in clonogenic assays by inflicting lethal DNA damage (e.g. DSBs) detected by immunofluorescence for γH2AX foci in the nucleus or by damaging the cell membrane. Preclinical studies of AE-emitting radiotherapeutic agents *in vivo* in mouse tumour xenograft models have further demonstrated that cancer treatment using AEs is feasible. Strong tumour growth inhibition has been achieved with minimal toxicity to normal tissues at the amounts administered due to the mostly restricted cytotoxicity of AEs towards cancer cells that bind these radiotherapeutic agents. A limited number of clinical studies of AEs for cancer therapy have been performed, and these mainly evaluated the tumour and normal tissue localisation of the radiotherapeutic agents, estimated normal organ dosimetry and assessed normal tissue toxicity at relatively low administered amounts. Nonetheless, some studies have shown promising results for treatment of cancer with AE-emitting radiotherapeutic agents (e.g. ^125^I-IUdR, ^111^In-DTPA-octreotide or ^125^I-mAb 425), achieving tumour remissions or improved survival in patients. The recent introduction of a large armamentarium of biologically-targeted therapies for cancer, especially humanised and fully human mAbs creates many new opportunities to design novel AE-emitting radiotherapeutic agents for cancer treatment. In only a few years, we will celebrate the 100th anniversary of the publication by Pierre Auger (Auger 1923) on the discovery of these electrons that bear his name. Pierre Auger did not conceive of the application of AEs for targeted cancer treatment, but this is a tremendously exciting future that we and many other scientists in this field envision.

## Data Availability

Not applicable.

## Abbreviations

^123^I-ITdU: ^123^I-4′-thio-2′-deoxyuridine
^123^I-MIBG: ^123^I-metaiodobenzylguanidine
^125^I-IUdR: ^125^I-iododeoxyuridine
^131^I-IUdR: ^131^I-iododeoxyuridine
^3^H-TdR: ^3^H-deoxyuridine
AEs: Auger electrons
AML: Acute myeloid leukemia
ATM: Ataxia telangiectasia mutated
AUC: Area under the curve
AuNPs: Gold nanoparticles
BC: Breast cancer
BCMs: Block copolymer micelles
BM: Bone marrow
bsRICs: Bispecific radioimmunoconjugates
CEA: Carcinoembryonic
CHO: Chinese Hamster Ovary
CI: Confidence interval
CS: Clonogenic survival
DMSO: Dimethylsulfoxide
DOTA: 1,4,7,10-tetraazacyclododecane-1,4,7,10-tetraacetic acid
DSBs: Double strand breaks
DTPA: Diethylenetriaminepentaacetic acid
EBRT: External beam radiotherapy
EC: Electron capture
EDDA: Ethylene diamine N,N′-diacetic acid
EGFR: Epidermal growth factor receptor
GBM: Glioblastoma multiforme
HAMA: Human anti-mouse antibodies
hEGF: Human epidermal growth factor
HER2: Human epidermal growth factor receptor 2
HMPAO: Hexamethylpropylenamineoxime
HYNIC: Hydrazinonicotinamide
i.p.: Intraperitoneal
i.t.: Intratumoural
IAEA: International Atomic Energy Agency
IC: Internal conversion
IUdR: ^125^I- or ^123^I-5-iodo-2-deoxyuridine
LET: Linear energy transfer
LSCs: Leukemic stem cells
mAb: Monoclonal antibody
MCPs: Metal-chelating polymers
MDR: Multidrug resistance
MIRD: Medical Internal Radiation Dose
MNTs: Modular nanotransporters
MPS: Mononuclear phagocyte system
NLS: Nuclear localisation sequence
NOAEL: No observable adverse effects
NOTA: 1,4,7-triazacyclononane-1,4,7-triacetic acid
NRG: NOD-*Rag1*^*null*^ *IL2rg*^*null*^, NOD rag gamma
NURBS: Non-uniform rational B-splines
PAMAM: Polyamidoamine
PCL: Polycaprolactone
PEG: Polyethylene glycol
PEG_24_: 24 mer PEG
PFGE: Pulsed-field gel electrophoresis
PRRT: Peptide receptor radionuclide therapy
p-SCN-Bn: p-isothiocyanatobenzyl
RaBiT: Radiotracer Biodistribution Template
RBE: Relative biological effectiveness
RICs: Radioimmunoconjugates
RIT: Radioimmunotherapy
ROS: Reactive oxygen species
r_S_: Source region
r_T_: Target region
SA: Specific activity
SCGE: Single cell gel electrophoresis assay
SD: Stable disease
SPECT: Single-photon emission computed tomography
sst-2: Somatostatin receptor subtype-2
T_b_: Biological half life
T_D_: Dose-integration period
T_e_: Effective half life
TMZ: Temozolomide
T_p_: Physical half-life
WBC: White blood cell
